# Thermal Expansion of Metal–Organic Framework Crystal–Glass Composites

**DOI:** 10.1021/acs.inorgchem.2c02663

**Published:** 2022-11-08

**Authors:** Christopher W. Ashling, Giulio I. Lampronti, Thomas J. F. Southern, Rachel C. Evans, Thomas D. Bennett

**Affiliations:** † Department of Materials Science and Metallurgy, University of Cambridge, CambridgeCB3 0FS, U.K.; ‡ Department of Earth Sciences, University of Cambridge, CambridgeCB2 3EQ, U.K.

## Abstract

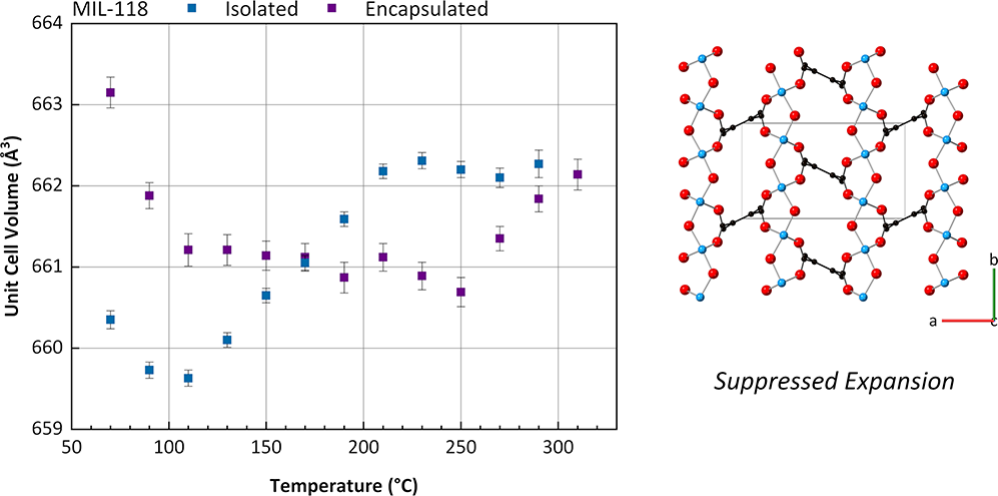

Metal–organic framework crystal–glass composites (MOF CGCs) are a class of materials comprising a crystalline framework embedded within a MOF glass matrix. Herein, we investigate the thermal expansion behavior of three MOF CGCs, incorporating two flexible (MIL-53(Al) and MIL-118) and one rigid (UL-MOF-1) MOF within a ZIF-62 glass matrix. Specifically, variable-temperature powder X-ray diffraction data and thermomechanical analysis show the suppression of thermal expansivity in each of these three crystalline MOFs when suspended within a ZIF-62 glass matrix. In particular, for the two flexible frameworks, the average volumetric thermal expansion (β) was found to be near-zero in the crystal–glass composite. These results provide a route to engineering thermal expansivity in stimuli-responsive MOF glass composites.

## Abstract 2

Metal−organic framework crystal−glass composites (MOF CGCs) are a novel class of composite materials that comprise crystalline MOFs within MOF glass matrices. In this work, we take a first glimpse at the expansion behavior of three separate crystalline MOFs within their respective MOF CGCs. It is observed that the surrounding glass matrix suppresses the expansion of each material leading to near-zero thermal expansion in two systems over a considerable temperature region.

## Introduction

Metal–organic frameworks (MOFs) are a class of hybrid materials, defined by the IUPAC as “a coordination network with organic ligands containing potential voids”.^1^ Their chemical and physical properties have garnered intense interest for potential applications such as molecular separation, catalysis, and sensing.^2−5^

The fabrication of bulk, contiguous materials composed partly or wholly of a MOF component is of great importance to industry, given the need for morphologies other than microcrystalline powders for application. Progress has been made in the fabrication of free-standing binder-free MOF monoliths through spark-plasma sintering^6^ and sol–gel processes,^7−9^ though most research involves supporting crystalline MOFs on a variety of substrates such as polymers, activated carbons, and silicas.^10^ Bulk structures have been formed through quenching the high-temperature liquid states of several zeolitic–imidazolate frameworks (ZIFs), a subset of MOFs characterized by their incorporation of imidazolate-based linkers in zeolitic architectures. For example, the ZIF-62 system, Zn(Im)_2–*x*_(bIm)*_x_* [Im, imidazolate; bIm, benzimidazolate], melts in the range 372–441 °C and, upon cooling, forms glasses with glass-transition temperatures (*T*_g_’s) in the range of 298–322 °C.^11−15^ The glasses, which contain tetrahedral metal nodes linked in a continuous random network by the imidazolate linkers, have demonstrated porosity to analyte gases from homodiatomic molecules such as hydrogen and nitrogen to gases as large as small-chain hydrocarbons such as propene.^16^ However, for molecules similar in size to propene, considerable diffusion limitations are observed.^16^

The ZIF-62 glass, denoted as *a*_g_ZIF-62, is of interest due to a wide range of properties, for example, high optical transmittance (∼90%) in the visible and near-infrared regions (*i.e*., 400–1600 nm). The refractive index (1.56 at 589 nm) and Abbe number, ν (*ca*. 31), of *a*_g_ZIF-62 place its optical properties in a comparable region of the refractive index–Abbe number diagram to the upper range of polymers.^17^ The incorporation of cobalt centers into *a*_g_ZIF-62 results in a mixed-metal zinc-cobalt analogue with nonlinear optical properties.^18^ Moreover, the mechanical properties of ZIF-glasses, in general, have been shown to exhibit characteristics of both inorganics and organics and exhibit resistance to ductile fracture.^14,19^

Such properties make *a*_g_ZIF-62 an attractive host matrix for a crystalline MOF component as the porosity and rigidity of the host matrix enables the preparation of bulk composite materials without compromising the functionality of the guest, crystalline MOF. Accordingly, several composite materials have been formed by mixing crystalline ZIF-62 with a crystalline MOF powder and heating the mixture to bring the ZIF-62 into the liquid state.^20,21^ After quenching, the resultant self-supporting bulk material comprises a well-dispersed crystalline MOF within the *a*_g_ZIF-62 matrix. These materials are referred to as metal–organic framework crystal–glass composites (MOF CGCs) and are denoted as (*crystal*)_x_(*glass*)_1–*x*_, where *x* is the weight fraction of the crystalline material in the composite, consistent with prior nomenclature.^20^

Owing to the relatively high melting temperatures (*T*_m_’s) of known glass-forming MOFs, only three MOF CGC systems have been formed via this route, all of which utilize *a*_g_ZIF-62 as the host matrix. The crystalline MOFs used in these MOF CGCs are MIL-53 [Al(OH)(C_8_H_4_O_4_)],^22^ MIL-118 [Al_2_(OH)_2_(C_10_O_8_H_2_)],^23^ and UL-MOF-1 [Li_2_(C_12_H_6_O_4_)].^24^

**Figure 1:**
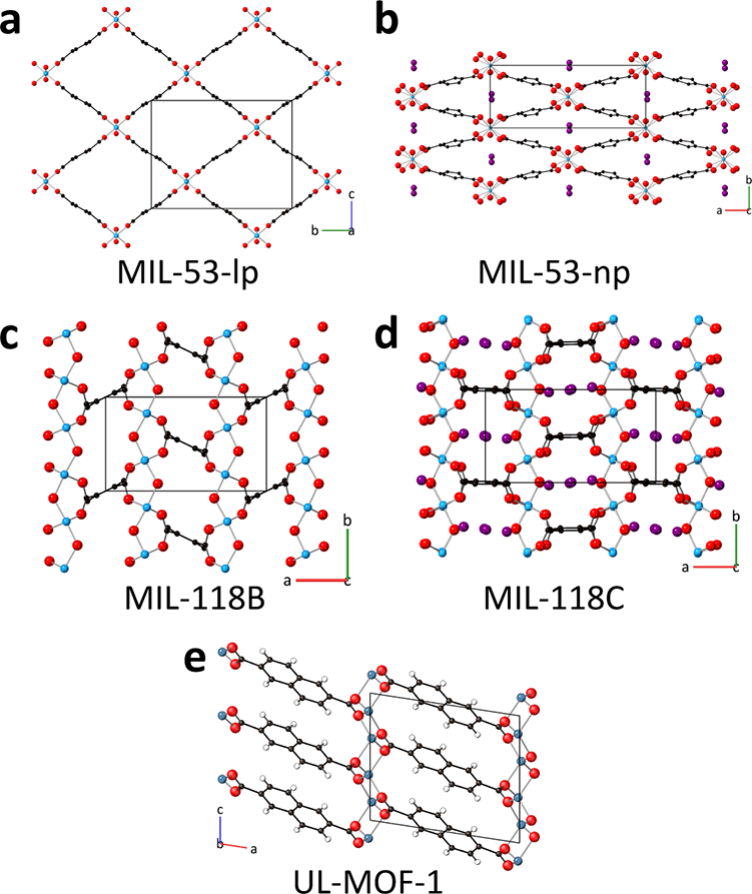
Crystal structure of (a) MIL-53-lp, (b) MIL-53-np, (c) MIL-118B, (d) MIL-118C (Al, blue; O, red; C, black; H, omitted for clarity), and (e) UL-MOF-1 (Li, blue; O, red; C, black; H, white). Unit cells are represented by black boxes.

MIL-53 and MIL-118 are members of the “breathing” framework family. These MOFs are typically synthesized with unreacted material occupying the pores of the framework, labeled MIL-53-as (orthorhombic, **Pnma**), and MIL 118A (monoclinic, **C*2/*c**), respectively. Upon activation of each framework through heating, the excess material is expelled, resulting in the formation of the high-temperature stable, open-pore phase of each material, labeled MIL-53-lp (orthorhombic, *Imma*), and MIL-118B (orthorhombic, *Pbam*), respectively. Upon cooling, these high-temperature phases spontaneously uptake atmospheric water to form the room-temperature-stable, activated phase, MIL-53-np (monoclinic, *Cc*) and MIL-118C (orthorhombic, *Pnam*), respectively. In this latter phase transition, the wine-rack-type pore structure of MIL-53 contracts, resulting in a considerable reduction framework pore volume (Figure 1a–b).^22^ However, this same phase transition MIL-118 results in a shift from the rectangular-shaped 1D tunnels in MIL-118B to lozenge-shaped channels in MIL-118C (Figure 1c–d).^23^ These two breathing frameworks may reversibly transition between their high-temperature and low-temperature states, through heating and cooling, respectively, expelling and readsorbing water in the process.

The third crystalline MOF, which has also been incorporated within *a*_g_ZIF-62, UL-MOF-1, is by contrast “rigid”. The structure comprises alternating antifluorite-type Li–O 2D layers connected by 2,6-naphthalenedicarboxylate (2,6-NDC) ligands and displays exceptional thermal stability (up to 610 °C) (Figure 1e).^24^

Though progress has been made on expanding the scope of possible MOF CGC materials through the use of novel fabrication methods, there remains little information regarding the effect of encapsulation on the physical properties of the crystalline MOF.^20,25^ Unusual physical behavior has been observed in the (MIL-53)*_x_*(*a*_g_ZIF-62)_1–*x*_ system, where the metastable open-pore MIL-53-lp phase is retained at room temperature; this phenomenon has been exploited to create MOF CGCs with significantly higher CO_2_ sorption capabilities.^26^ In contrast, while MIL-118 is also a “breathing” framework, the open-pore MIL-118B phase is not stabilized at room temperature in the composite, and the MIL-118C phase is observed in the MOF CGC. The behavioral divergence of the crystalline components in these two systems demonstrates that the nature of the fabricated MOF CGCs is more complex than that of a noninteracting system.

One approach to investigating intracomposite interactions is to probe the response of the composite to thermal stimulus. The calculated volumetric or uniaxial response to temperature change is known as thermal expansivity. This property may be studied on a macroscopic (direct sample measurement) or microscopic (unit cell) scale, each with distinct advantages and sensitivities.^27^ In addition, understanding the material’s thermal behavior is critical for determining its application in dynamic temperature systems. This is not only an essential practical consideration in applied settings but may also impact the material chemistry as alteration of the size and shape of the pores has direct implications on the host–guest interaction strength. While the expansion behaviors of *a*_g_ZIF-62, MIL-53, Na_2_NDC (a sodium analogue of UL-MOF-1), and MIL-118 have all been probed separately, there exists no study detailing the unit cell expansion of any crystalline material within a MOF glass.^21,23,28,29^ Motivated by the absence of prior studies, here, we compare and contrast the change in thermal expansivity of three crystalline MOFs upon encapsulation within a MOF CGC.

## Experimental Section

### Synthesis of MIL-53

The same synthetic procedure as reported in ref (22) and activation from ref (16) were followed. Specifically, aluminum nitrate nonahydrate (26 g, 6.93 × 10^–2^ mol) and terephthalic acid (5.76 g, 4.96 × 10^–2^ mol) were dissolved in water (100 mL) and put into a Teflon-lined autoclave and placed in an oven at 220 °C for 72 h. The resulting powder was washed with deionized water (3 × 30 mL) and dried in a vacuum oven at 150 °C for 24 h. MIL-53 was activated by heating at 330 °C for 72 h, and then to 450 °C for 6 min before cooling to room temperature (RT).

### Synthesis of ZIF-62

The same synthetic procedure as reported in refs^15^ and (16) was followed. Specifically, zinc nitrate hexahydrate (1.65 g, 5.54 × 10^–3^ mol) and imidazole (8.91 g, 0.13 mol) were added to a 200 mL screw-top jar, dissolved in *N*,*N*-dimethylformamide (DMF, 75 mL) and stirred for 1 h. Once complete dissolution was achieved, benzimidazole (1.55 g, 1.31 × 10^–2^ mol) was added and heated to 130 °C for 48 h. The product was allowed to cool to room temperature, and crystals were separated by vacuum-assisted filtration and washed with DMF (40 mL) and dichloromethane (DCM, 40 mL) before being dried in the oven at 60 °C overnight.

### Synthesis of MIL-118

The same synthetic procedure as reported in refs (23) and (16) was followed. Specifically, aluminum nitrate nonahydrate (150 mg, 7.04 × 10^–4^ mol) and benzene-1,2,4,5-tetracarboxylic acid (50 mg, 1.97 × 10^–4^ mol) were added to a Teflon-lined autoclave before adding water (5 mL). The autoclave was sealed and placed into a 210 °C preheated oven for 24 h. The product of this was isolated by replacing the liquid with water (20 mL) and centrifuging (2500 rpm, 10 min) twice. The resultant white powder was placed in a 70 °C preheated oven overnight.

### Synthesis of UL-MOF-1

The same synthetic procedure as reported in refs (24) and (16) was followed. Specifically, lithium nitrate (0.345 g, 5.00 × 10^–3^ mol), naphthalene-2,6-dicarboxylic acid (0.565 g, 2.61 × 10^–3^ mol), ammonium fluoride (38 mg), and DMF (15 mL) were added to a Teflon-lined autoclave and placed in a 180 °C preheated oven and held for 5 days. Upon cooling, the reaction mixture was transferred to a centrifuge tube and the liquid was replaced with ethanol (20 mL). The sample was stirred for 5 min before centrifuging (3000 rpm, 5 min) to collect a white powder, which was dried in an oven at 60 °C overnight.

### Synthesis of MOF CGC Materials

The same synthetic procedure as reported in ref (16) was followed. Specifically, ZIF-62 and the corresponding crystalline material were ball-milled together using a Retsch MM400 instrument, in appropriate wt % ratios using a 7 mm diameter stainless steel ball for 15 min, at a frequency of 30 Hz. The mixed powder was pressed in a 13-mm-diameter die at 0.74 GPa for 1 min. The pellet was then clamped between glass slides, heated to 450 °C in a tube furnace at a rate of 20 °C/min under an Ar atmosphere, and held for 15 min before being cooled to RT.

### Thermomechanical Analysis

Data were taken on a small portion of each of the as-synthesized composite monolith materials on a TA Instruments Q400 thermomechanical materials analyzer (TMA). The experiment was performed with a force of 0.05 N and protected by N_2_ gas.

### Variable-Temperature Powder X-ray Diffraction

Each material was mixed with ∼10% by volume of silicon powder (Si) and ground together using a mortar and pestle. Data were collected using a Bruker D8 Advance equipped with an MRI high-temperature chamber and a Vantec detector, using Cu Kα radiation (λ = 1.5418 Å) under vacuum (8.5 × 10^–3^ mbar). Prior to running the experiments, height adjustments were performed to optimize the full width at half-maximum (FWHM) of the (111) silicon standard reflection at 2θ ∼ 28.45°. Samples were heated in 20 °C increments from 30 °C to an appropriate end temperature. Diffraction patterns at 2θ values of 5–40° were recorded at each increment with a time/step of 0.6 s over 0.04° steps.

### PXRD Data Refinement

Data were analyzed with TOPAS academic (V6) software.^30,31^ Reported thermal expansion data for Si provided an accurate calculation of unit cell parameters for the Si standard.^32^ Using these values, peak position was corrected for sample displacement across sample series. XRD data were refined sequentially using the reported crystallographic information files of MIL-118B, MIL-53-lp, or UL-MOF-1; atomic positions were included but were constrained in the refinements.^22−24^ To account for the diffuse scattering from *a*_g_ZIF-62 in the MOF CGCs, a broad Gaussian peak was added and permitted to refine sequentially. Subsequent refinements in the series were performed using the final values for the previous pattern as initial values. A Pearson VII function and an eighth-order Chebychev polynomial background were used to model the peak shape and the background, respectively. Scale factors, unit cell parameters, and eighth-order spherical harmonics for preferred orientation corrections were refined individually for all scans.

## Results and Discussion

Samples of three previously reported MOF CGCs, (MIL-53)_0.25_(*a*_g_ZIF-62)_0.75_, (MIL-118)_0.5_(*a*_g_ZIF-62)_0.5_, and (UL-MOF-1)_0.5_(*a*_g_ZIF-62)_0.5_, were synthesized according to previously published procedures (see the Experimental Section, Figures S1–S3).^23,24^ In brief, the CGCs were formed by ball-milling the glass-former (ZIF-62) and the nonmelting MOF together before heating to 450 °C for 15 min and cooling to room temperature. The recorded powder X-ray diffraction (PXRD) data for the synthesized MOF CGCs were consistent with previously reported data (Figures S1–S6).^16,20^ Bragg peaks emerging from the amorphous background of the *a*_g_ZIF-62, corresponding to UL-MOF-1, the large-pore MIL-53-lp phase, and the low-temperature MIL-118C phase, were observed in the respective MOF CGCs.

**Figure 2:**
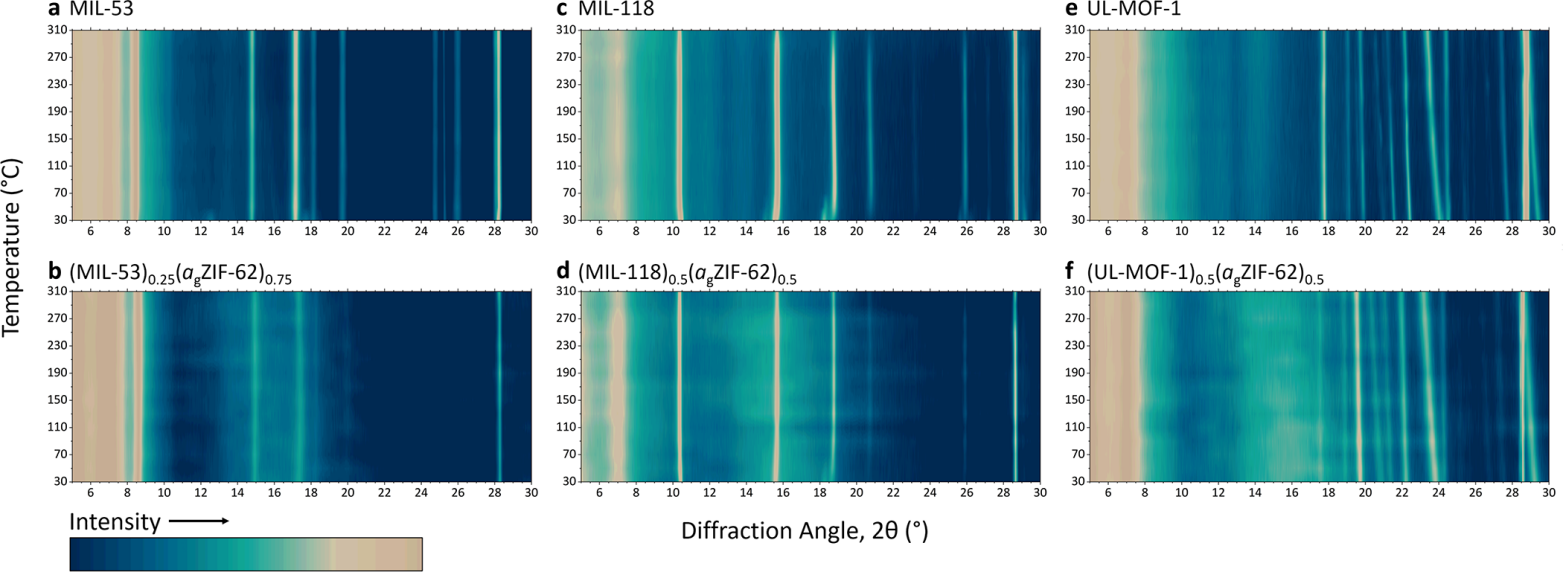
Contour maps of variable-temperature powder X-ray diffraction data for (a) MIL-53, (b) (MIL-53)_0.25_(*a*ZIF-62)_0.75_, (c) MIL-118, (d) (MIL-118)_0.5_(*a*ZIF-62)_0.5_, (e) UL-MOF-1, and (f) (UL-MOF-1)_0.5_(*a*ZIF-62)_0.5_. Intensity scale bar below, of which units are arbitrary.

Variable-temperature PXRD (VT-PXRD) was carried out to study the unit cell expansion of the three crystalline samples and their respective MOF CGCs. Samples of MIL-53-np, (MIL-53)_0.25_(*a*_g_ZIF-62)_0.75_, MIL-118, **(**MIL-118)_0.5_(*a*_g_ZIF-62)_0.5_, UL-MOF-1, and (UL-MOF-1)_0.5_(*a*_g_ZIF-62)_0.5_, were doped with a silicon standard (approximately 10% by volume), flattened onto a sample holder, and placed under vacuum (8.5 × 10^–3^ mbar). The sample displacement was then corrected using an internal standard (Si, see the Experimental Section). Each experiment began by heating the sample to 30 °C and equilibrating for 5 min before recording the initial PXRD pattern. Data were subsequently collected at 20 °C intervals to 310 °C, allowing for thermal equilibration before each collection (Figures 2 and S7–S12). Unit cell parameters of the crystalline materials were then extracted using Rietveld refinement of the VT-PXRD data (see the Experimental Section). The temperature-dependent expansion values of the refined unit cells were calculated as follows:
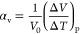
1where α_ν_ is the volumetric coefficient of thermal expansion (CTE) and *V* is the cell volume.^27^ The mean value of Δ*V*/Δ*T* may be determined by extracting the gradient from a linear region of a volume-temperature plot or differentiating a second-order polynomial fit. Similarly, the linear CTE, α_a_, may be determined from the change in each unit cell parameter as
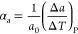
2

This equation is valid for materials that (i) exhibit small changes in the CTE over the measured temperature range and (ii) undergo small expansion values relative to the initial volume of the material (see eqs S1–S3). These assumptions are valid for all crystalline MOFs measured here. The volumetric and linear CTEs for the isolated MOFs and crystalline MOFs within the MOF CGCs were calculated (Tables 1 and S1–S6).

**Table 1:** Volumetric and Linear Coefficients of Unit Cell Thermal Expansion. Errors Are Given as the Average Standard Deviation, Reported to 5 sf

		volumetric	linear		
sample	temperature range (°C)	α_ν_^a^ (10^–6^ K^–1^)	α_a_^a^ (10^–6^ K^–1^)	α_b_^a^ (10^–6^ K^–1^)	α_c_^a^ (10^–6^ K^–1^)
MIL-118B	110–230	35.572(5)	51.795(6)	–3.1281(2)	–12.982(1)
	230–290	–1.6609(3)	–8.8327(8)	–3.8524(2)	10.795(1)
(MIL-118)_0.5_(*a*_g_ZIF-62)_0.5_	110–250	–4.9963(15)	–2.9865(7)	–4.0969(7)	2.0710(2)
	250–310	36.628(10)	30.507(6)	4.8333(7)	1.1480(1)
UL-MOF-1	30–310	117.84(5)	0.84381(20)	18.300(5)	90.226(22)
(UL-MOF-1)_0.5_(*a*_g_ZIF-62)_0.5_	30–310	103.22(7)	–3.3784(19)	16.398(3)	80.862(24)
MIL-53-lp	70–310	2.2259(23)	1.9377(6)	–6.3306(54)	6.6444(34)
(MIL-53)_0.25_(*a*_g_ZIF-62)_0.75_	70–310	5.3364(123)	–4.1684(26)	6.9688(133)	2.5676(29)

a Single value using the lowest temperature of the specified temperature range.

**Figure 3:**
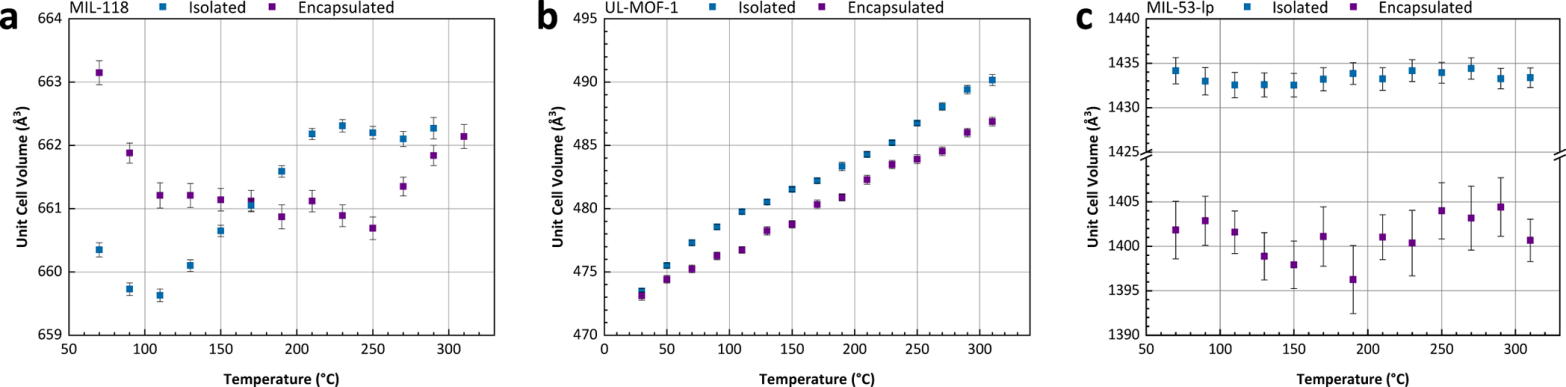
Refined unit cell volumes of the isolated MOFs and crystal–glass composites of (a) MIL-118, (b) UL-MOF-1, and (c) MIL-53-lp. Estimated standard deviations are shown as error bars.

Three distinct regions of unit cell volume change for the crystalline MIL-118 sample are evident in Figure 3a. The initial decrease in unit cell volume up to 110 °C may be attributed to the contraction of the structure upon conversion from MIL-118C to MIL-118B as water is expelled from the framework. Evidence of this conversion is apparent from the change in PXRD pattern as shown in Figure 2, and Figure S9, most notably the peak at *ca.* 18° 2θ. On completion of the transformation to MIL-118B, the structure expands uniformly between 110 and 230 °C where the first region of α_ν_ is calculated (35.6 × 10^–6^ K^–1^, taken at 110 °C). The expansion over this range is dominated by extension along the *a* axis (α_a_ = 51.8 × 10^–6^ K^–1^), which details the distance between Al–O columns connected by ortho-substituted carboxylates around the benzene-1,2,4,5-tetracarboxylate linkers. Above 230 °C, negligible change in the unit cell volume is observed (α_ν_ = −1.66 × 10^–6^ K^–1^, taken at 230 °C), possibly demonstrating a maximum unit cell volume—and by extension, pore size—under the experimental conditions. Decomposition of the sample is evident from the peak intensity reduction toward the end of the experiment (Figure S9); data at 310 °C are therefore omitted from the calculations.

**Figure 4:**
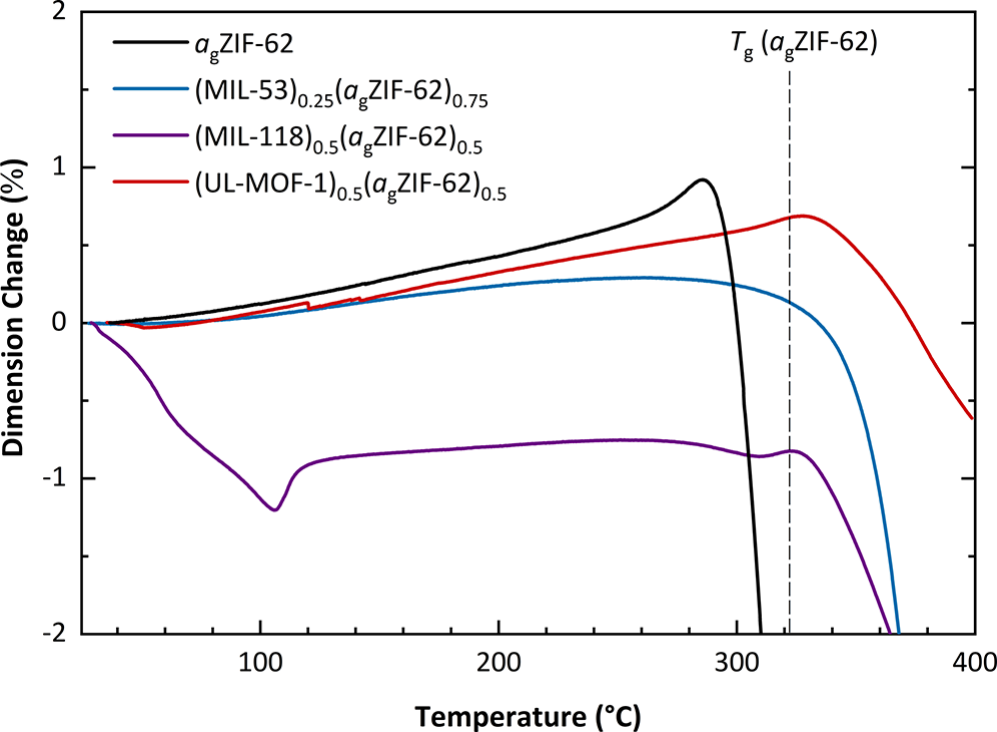
Thermomechanical analysis (TMA) of (MIL-53)_0.25_(*a*_g_ZIF-62)_0.75,_ (MIL-118)_0.5_(*a*_g_ZIF-62)_0.5_, and (UL-MOF-1)_0.5_(*a*_g_ZIF-62)_0.5_, including guideline for the largest reported *T*_g_ of *a*_g_ZIF-62.^15^

The unit cell expansion of MIL-118 within (MIL-118)_0.5_(*a*_g_ZIF-62)_0.5_ is also observed to undergo three distinct regions of change. The first region is analogous to the isolated material, where unit cell contraction occurs during the conversion to the MIL-118B phase, ending at 110 °C. After 110 °C, the thermal behavior of the encapsulated MIL-118B diverges from the isolated sample; rather than steadily expanding, a slight decrease in the unit cell volume is observed from 110 to 250 °C (α_ν_ = −5.00 × 10^–6^ K^–1^, taken at 110 °C). At 250 °C, MIL-118B begins expanding at a similar rate (α_ν_ = 36.6 × 10^–6^ K^–1^, taken at 250 °C) to the expanding region of the isolated crystalline material. The temperature at which MIL-118B begins to expand within *a*_g_ZIF-62 is broadly comparable to the softening point of *a*_g_ZIF-62, as demonstrated in the thermomechanical analysis in Figure 4. This thermal behavior may be caused by the suppression of MIL-118 expansion by the rigid glass matrix, which permits the material to expand as it softens.

The “rigid” UL-MOF-1 framework was observed to expand linearly across the 30–310 °C temperature range in this experiment via a single mode of expansion (Figure 3b, Tables S5 and S6). A single value of the volumetric CTE (α_ν_) of UL-MOF-1 from 30–310 °C was therefore calculated to be 118 × 10^–6^ K^–1^, which is dominated by expansion along the *b* and *c* axes (18.3 × 10^–6^ K^–1^ and 90.2 × 10^–6^ K^–1^, respectively). Connectivity along the *bc* plane aligns with the planes of the Li–O sheets that make up UL-MOF-1. Li–Li distances in this plane must, therefore, increase as the area expands. The expansivity along the *a* axis is almost negligible (1 × 10^–6^ K^–1^), which describes the distance between nearest lithium atoms on adjacent Li–O sheets and is limited by the length of the connecting 2,6-NDC linkers. These results are in accordance with a study carried out on a sodium analogue of UL-MOF-1, which details a decrease in the unit cell β angle and an increase in the volumetric and *b* parameters^29^

The expansion mode of UL-MOF-1 within (UL-MOF-1)_0.5_(*a*_g_ZIF-62)_0.5_ is identical to the isolated crystalline material; however, α_ν_ over the same temperature range was reduced by 12.4%, to 103 × 10^–6^ K^–1^ in the glass. It is apparent that expansion suppression by the *a*_g_ZIF-62 matrix is present even for MOF CGC systems containing “rigid” crystalline MOFs.

A sample of MIL-53-np was prepared through the calcination of MIL-53-as (see the Experimental Section). Upon reducing atmospheric pressure in the experimental setup, MIL-53-np underwent expansion to the MIL-53-lp phase, according to previous studies.^23^ Incomplete conversion at this stage was evidenced by the presence of small Bragg peaks corresponding to MIL-53-np present in the PXRD pattern recorded at 30 °C but were no longer present by 70 °C. Refinement of the patterns from 70 to 310 °C indicated no change within the error. The near-zero net thermal expansion of MIL-53-lp over this range is broadly consistent with the <0.3% volumetric expansion of MIL-53-lp observed in a previous study, where no pressure reduction was applied.^28^ This expansivity behavior is also observed for a sample of (MIL-53)_0.25_(*a*_g_ZIF-62)_0.75,_ which was present in the MIL-53-lp phase as a result of the fabrication method (Figure 3c).

Previous studies have demonstrated that the retention of MIL-53-lp in the MOF CGC is not a result of the *a*_g_ZIF-62 hydrophobicity, suppressing the water-driven narrowing of the pores.^20^ While linker penetration into the pores of the crystalline MOF remains a possibility for the retention of MIL-53-lp in the composite, this work supports an alternative explanation for the disparity in behavior between MIL-53 and MIL-118 based upon the volume expansion of the crystalline materials.

The melting and glass-transition temperatures of ZIF-62 are far greater than those required to convert MIL-53 or MIL-118 to their respective high-temperature phases. Therefore, in the process of forming a MOF CGC, ZIF-62 melts and subsequently flows. At this temperature, MIL-53 and MIL-118 are present in their high-temperature phases. The relatively high vitrification temperature of ZIF-62 (*T*_g_ > 293 °C) means that when the glass is formed, on cooling, the high-temperature phases of MIL-53 and MIL-118 are still present. This, therefore, excludes the possibility that the difference in behavior is simply due to the temperature of transitions.

Therefore, we propose the volume expansion as the reason why MIL-118 reverts to the low-temperature phase where MIL-53 does not. The transition from the high- to low- temperature phases of both MIL-53 and MIL-118 is accompanied by considerable volumetric change. However, it has been shown here that significant expansion of these crystalline materials is hindered within the glass phase. A possibility for why MIL-53 remains in the MIL-53-lp phase is due to the substantial uniaxial expansion (17.02%) upon cooling to the MIL-53-np phase (Table S7). While a perpendicular 40.59% contraction is also observed in the narrowing of the MIL-53 pores, the glass which has solidified around the MIL-53-lp phase may not be able to accommodate the expansion. However, in the case of MIL-118, the largest uniaxial expansion toward the MIL-118C phase is only 7.08% (Table S7). This difference in uniaxial expansion upon transition to the low-temperature phase may be the cause of the resultant phase behavior divergence in the MOF CGCs. Thus, the presence of MIL-53-lp at room temperature in the MOF CGC may be a result of physical obstruction by the denser MOF glass matrix.

### Bulk Measurements

Complementary to the study of the crystalline MOFs within the composite, the thermal expansion of the bulk composites was recorded using thermomechanical analysis (TMA). This method involves the application of a very small force (0.05 N in this case) to the surface of a material and measures the change in material length upon heating. Such an analysis provides a one-dimensional change in length, *L*, over the temperature, *T*; quantifying the change, Δ*L*/Δ*T* provides a length variant of the CTE, α_L_. The application of TMA allows for the determination of expansion for amorphous (glass) materials where VT-PXRD is unable. However, finding isotropic expansion values of microcrystalline powders may be prohibitively difficult using TMA. Since neither technique can provide an evaluation of both microscopic and macroscopic material expansion, this work notably employs both techniques in the assessment of MOF CGC bulk expansion properties.

Samples of *a*_g_ZIF-62, (MIL-53)_0.25_(*a*_g_ZIF-62)_0.75_, (MIL-118)_0.5_(*a*_g_ZIF-62)_0.5_, and (UL-MOF-1)_0.5_(*a*_g_ZIF-62)_0.5_ were thus probed using TMA. Experimental data are recorded in absolute length, so for meaningful comparison of material expansivity, data reported here are in percentage dimension change to account for differences in initial length (Figure 4).

A pure sample of *a*_g_ZIF-62 is observed to exhibit the largest thermal expansivity (α_L_ = 32.11 × 10^–6^ K^–1^) of the measured materials, following previously reported data (35 × 10^–6^ K^–1^).^21^ Predictably, the sample of (UL-MOF-1)_0.5_(*a*_g_ZIF-62)_0.5_ with the largest volumetric expansion also exhibits the largest thermal expansion of the measured composites (α_L_ = 27.59 × 10^–6^ K^–1^, 143–306 °C). A smaller expansion of (MIL-53)_0.25_(*a*_g_ZIF-62)_0.75_ (α_L_ = 14.22 × 10^–6^ K^–1^, 111–177 °C) is likely due to the very small expansion of the MIL-53-lp phase inside the composite, and a larger contributing volume of ZIF-62 compared to that in (UL-MOF-1)_0.5_(*a*_g_ZIF-62)_0.5_. The initial sharp decrease in length in the (MIL-118)_0.5_(*a*_g_ZIF-62)_0.5_ is ascribed to the temperature-induced phase change of MIL-118 from MIL-118C to MIL-118B as observed in VT-PXRD. After this phase change, a small thermal expansion (α_L_ = 8.79 × 10^–6^ K^–1^, 128–270 °C) is observed, arising from the combination of the negative thermal expansion from composited MIL-188B and the positive thermal expansion of *a*_g_ZIF-62.

The density of each metal–organic framework (*a*_g_ZIF-62 = ∼1.57 g cm^–3^, MIL-118B = 1.696 g cm^–3^, UL-MOF-1 = 1.606 g cm^–3^, and MIL-53-lp = 0.9797 g cm^–3^)^11,22−24^ is accounted for by assuming that the contribution of each material to the predicted CTE is equivalent to its vol %. An “isotropic” value of one-dimensional expansivity, calculated by the average over the three mutually perpendicular coordinate axes (as determined by VT-PXRD), may represent the crystalline MOF contribution to the 1D bulk expansivity. Averaging these isotropic values along with the measured value of *a*_g_ZIF-62, weighted by their volume contributions, provides a predicted expansivity of a noninteracting system.

The calculated value of (UL-MOF-1)_0.5_(*a*_g_ZIF-62)_0.5_ using data from the encapsulated UL-MOF-1 is nearer to the measured value than using the isolated crystalline UL-MOF-1 data. Samples of (MIL-53)_0.25_(*a*_g_ZIF-62)_0.75_ and (MIL-118)_0.5_(*a*_g_ZIF-62)_0.5_, however, show an appreciable reduction in expansivity compared to the calculated values. While crystalline expansion values are reliably calculated from VT-PXRD refinements, these calculations assume that no substantial preferred crystalline orientation is induced during the synthesis of the MOF CGCs. The range of α_L_ value boundaries under extreme orientation conditions are hence calculated by substituting the averaged, “isotropic” CTE value for the minimum and maximum linear CTE values of each crystalline material within the composite (Table 2).

**Table 2:** Calculated and Measured 1D Expansion of the MOF CGCs Studied Herein

			aligned crystalline orientation		
	weighted combination of isolated MOF components (10^–6^ K^–1^)	weighted combination of components within the MOF CGC (10^–6^ K^–1^)	minimum value (10^–6^ K^–1^)	maximum value (10^–6^ K^–1^)	measured (10^–6^ K^–1^)
*a*_g_ZIF-62					32.11
(MIL-53)_0.25_(*a*_g_ZIF-62)_0.75_	21.18	21.54	19.47	23.35	14.22
(MIL-118)_0.5_(*a*_g_ZIF-62)_0.5_	22.37	15.87	14.70	17.67	8.79
(UL-MOF-1)_0.5_(*a*_g_ZIF-62)_0.5_	36.19	33.44	16.65	60.84	27.59

The range of possible (UL-MOF-1)_0.5_(*a*_g_ZIF-62)_0.5_ CTE values is relatively vast. However, the predicted and measured values remain broadly comparable, signifying no great degree of preferred orientation. In contrast, for the composites with “flexible” crystalline MOFs, (MIL-53)_0.25_(*a*_g_ZIF-62)_0.75_, and (MIL-118)_0.5_(*a*_g_ZIF-62)_0.5_, the near-zero CTE values along each crystallographic axis acutely narrow the range of values in extreme conditions. Notably, for these samples, the measured data remain considerably out of the predicted range. While preferential orientation may affect the measured data, it does not fully account for the disparity in predicted and measured values, even accounting for the minimum expansivity of the flexible crystalline MOF. Two further compounding factors may be (i) a discrepancy between the expansion of *a*_g_ZIF-62 in the pure and composite samples and (ii) macrostructural features, such as interfacial void spaces, that cause deviation in recorded values. If the former is true, a reduction in expansivity of *a*_g_ZIF-62 may indicate interacting behavior between the *a*_g_ZIF-62 and the composited crystalline MOF.

## Conclusions

In this work, the effect on the unit cell expansion of three crystalline MOFs, when suspended within an *a*_g_ZIF-62 matrix, was analyzed using refinements of VT-PXRD data. Bulk expansivity measurements were then recorded for *a*_g_ZIF-62 and all three MOF CGCs using TMA. Having determined the expansivity of the encapsulated crystalline materials and an isolated sample of *a*_g_ZIF-62, the one-dimensional bulk expansivity of the MOF CGCs was approximated using a weighted average of the component materials. A comparison of these values with recorded data for the MOF CGCs was performed to validate the approximation and speculate on possible MOF–*a*_g_MOF interactions.

When encapsulated within *a*_g_ZIF-62, the unit cell volume thermal expansivity of UL-MOF-1 behaves similarly to the pure crystalline material but is reduced by 12.4%. In contrast, samples of (MIL-53)_0.25_(*a*_g_ZIF-62)_0.75_ and (MIL-118)_0.5_(*a*_g_ZIF-62)_0.5_ display near-zero volumetric thermal expansion of the crystalline MOFs. As a result, the aperture size of these flexible frameworks remains relatively stable within *a*_g_ZIF-62 compared to their isolated crystalline materials. The fixture of these apertures may be key to the reliability of host–guest interactions for systems utilizing MIL-53 or MIL-118 over the measured temperature ranges.

The experiments herein support an argument that the degree of expansion of the flexible crystalline component upon returning to the low-temperature phase determines whether the high-temperature phase is present in the room-temperature composite. Therefore, it is proposed that metastable high-temperature phases of flexible systems with significant uniaxial expansion on cooling may be retained within a MOF CGC.

Furthermore, bulk expansivity approximations using a combination of VT-PXRD and TMA data were shown to greatly overestimate values for samples of (MIL-53)_0.25_(*a*_g_ZIF-62)_0.75_ and (MIL-118)_0.5_(*a*_g_ZIF-62)_0.5_, which may be a result of MOF–*a*_g_MOF chemical interactions. The development of bulk property predictions presents an opportunity to produce zero thermal expansion MOF CGCs by offsetting the expansivity of the glass through the incorporation of MOFs with negative thermal expansivities.^33^ Such materials may be useful for precision equipment such as mirror substrates and laser gyros where shape and length consistency are required.^34^

## Supporting Information Available

Initial PXRD refinement with difference and hkl tick marks of each sample, stacked VT-PXRD with overlaid refinement, tabulated refinement data, and evidenced Taylor expansion for the coefficient of thermal expansion (PDF)

## References

[ref1] Batten S. R.; Champness N. R.; Chen X.-M.; Garcia-Martinez J.; Kitagawa S.; Öhrström L.; O’Keeffe M.; Suh M. P.; Reedijk J. Terminology of Metal–Organic Frameworks and Coordination Polymers (IUPAC Recommendations 2013)*. Pure Appl. Chem. 2013, 85, 1715–1724. 10.1351/PAC-REC-12-11-20.

[ref2] Lin Z. T.; Liu Q. Y.; Yang L.; He C.-T.; Li L.; Wang Y.-L. Fluorinated Biphenyldicarboxylate-Based Metal-Organic Framework Exhibiting Efficient Propyne/Propylene Separation. Inorg. Chem. 2020, 59, 4030–4036. 10.1021/acs.inorgchem.0c00003. 32118410

[ref3] Goetjen T. A.; Liu J.; Wu Y.; Sui J.; Zhang X.; Hupp J. T.; Farha O. K. Metal-Organic Framework (MOF) Materials as Polymerization Catalysts: A Review and Recent Advances. Chem. Commun. 2020, 56, 10409–10418. 10.1039/d0cc03790g. 32745156

[ref4] Kreno L. E.; Leong K.; Farha O. K.; Allendorf M.; Van Duyne R. P.; Hupp J. T. Metal-Organic Framework Materials as Chemical Sensors. Chem. Rev. 2012, 112, 1105–1125. 10.1021/cr200324t. 22070233

[ref5] Vikrant K.; Tsang D. C. W.; Raza N.; Giri B. S.; Kukkar D.; Kim K. H. Potential Utility of Metal-Organic Framework-Based Platform for Sensing Pesticides. ACS Appl. Mater. Interfaces 2018, 10, 8797–8817. 10.1021/acsami.8b00664. 29465977

[ref6] Widmer R. N.; Lampronti G. I.; Kunz B.; Battaglia C.; Shepherd J. H.; Redfern S. A. T.; Bennett T. D. Manufacturing Macroporous Monoliths of Microporous Metal–Organic Frameworks. ACS Appl. Nano Mater. 2018, 1, 497–500. 10.1021/acsanm.7b00335.

[ref7] Tian T.; Velazquez-Garcia J.; Bennett T. D.; Fairen-Jimenez D. Mechanically and Chemically Robust ZIF-8 Monoliths with High Volumetric Adsorption Capacity. J. Mater. Chem. A 2015, 3, 2999–3005. 10.1039/c4ta05116e.

[ref8] Tian T.; Zeng Z.; Vulpe D.; Casco M. E.; Divitini G.; Midgley P. A.; Silvestre-Albero J.; Tan J. C.; Moghadam P. Z.; Fairen-Jimenez D. A Sol-Gel Monolithic Metal-Organic Framework with Enhanced Methane Uptake. Nat. Mater. 2018, 17, 174–179. 10.1038/NMAT5050. 29251723

[ref9] Connolly B. M.; Madden D. G.; Wheatley A. E. H.; Fairen-Jimenez D. Shaping the Future of Fuel: Monolithic Metal–Organic Frameworks for High-Density Gas Storage. J. Am. Chem. Soc. 2020, 142, 8541–8549. 10.1021/jacs.0c00270. 32294384

[ref10] Zhu Q.-L.; Xu Q. Metal–Organic Framework Composites. Chem. Soc. Rev. 2014, 43, 5468–5512. 10.1039/c3cs60472a. 24638055

[ref11] Bennett T. D.; Yue Y.; Li P.; Qiao A.; Tao H.; Greaves N. G.; Richards T.; Lampronti G. I.; Redfern S. A. T.; Blanc F.; Farha O. K.; Hupp J. T.; Cheetham A. K.; Keen D. A. Melt-Quenched Glasses of Metal-Organic Frameworks. J. Am. Chem. Soc. 2016, 138, 3484–3492. 10.1021/jacs.5b13220. 26885940

[ref12] Frentzel-Beyme L.; Kloß M.; Pallach R.; Salamon S.; Moldenhauer H.; Landers J.; Wende H.; Debus J.; Henke S. Porous Purple Glass-a Cobalt Imidazolate Glass with Accessible Porosity from a Meltable Cobalt Imidazolate Framework. J. Mater. Chem. A 2019, 7, 985–990. 10.1039/c8ta08016j.

[ref13] Frentzel-Beyme L.; Kloß M.; Kolodzeiski P.; Pallach R.; Henke S. Meltable Mixed-Linker Zeolitic Imidazolate Frameworks and Their Microporous Glasses: From Melting Point Engineering to Selective Hydrocarbon Sorption. J. Am. Chem. Soc. 2019, 141, 12362–12371. 10.1021/jacs.9b05558. 31288513

[ref14] Li S.; Limbach R.; Longley L.; Shirzadi A. A.; Walmsley J. C.; Johnstone D. N.; Midgley P. A.; Wondraczek L.; Bennett T. D. Mechanical Properties and Processing Techniques of Bulk Metal-Organic Framework Glasses. J. Am. Chem. Soc. 2019, 141, 1027 10.1021/jacs.8b11357. 30582804

[ref15] Qiao A.; Bennett T. D.; Tao H.; Krajnc A.; Mali G.; Doherty C. M.; Thornton A. W.; Mauro J. C.; Greaves G. N.; Yue Y. A Metal-Organic Framework with Ultrahigh Glass-Forming Ability. Sci. Adv. 2018, 4, eaao6827 10.1126/sciadv.aao6827. 29536040 PMC5844704

[ref16] Ashling C. W.; Macreadie L. K.; Southern T. J. F.; Zhang Y.; McHugh L. N.; Evans R. C.; Kaskel S.; Telfer S. G.; Bennett T. D. Guest Size Limitation in Metal-Organic Framework Crystal-Glass Composites. J. Mater. Chem. A 2021, 9, 8386–8393. 10.1039/d0ta11229a.

[ref17] Qiao A.; Tao H.; Carson M. P.; Aldrich S. W.; Thirion L. M.; Bennett T. D.; Mauro J. C.; Yue Y. Optical Properties of a Melt-Quenched Metal–Organic Framework Glass. Opt. Lett. 2019, 44, 1623 10.1364/ol.44.001623. 30933106

[ref18] Ali M. A.; Liu X.; Li Y.; Ren J.; Qiu J. Nonlinear-Optical Response in Zeolitic Imidazolate Framework Glass. Inorg. Chem. 2020, 59, 8380–8386. 10.1021/acs.inorgchem.0c00806. 32482059

[ref19] Stepniewska M.; Januchta K.; Zhou C.; Qiao A.; Smedskjaer M. M.; Yue Y. Observation of Indentation-Induced Shear Bands in a Metal-Organic Framework Glass. Proc. Natl. Acad. Sci. U.S.A. 2020, 117, 10149–10154. 10.1073/pnas.2000916117. 32341165 PMC7229652

[ref20] Hou J.; Ashling C. W.; Collins S. M.; Krajnc A.; Zhou C.; Longley L.; Johnstone D. N.; Chater P. A.; Li S.; Coulet M.-V.; Llewellyn P. L.; Coudert F.-X.; Keen D. A.; Midgley P. A.; Mali G.; Chen V.; Bennett T. D. Metal-Organic Framework Crystal-Glass Composites. Nat. Commun. 2019, 10, 2580 10.1038/s41467-019-10470-z. 31189892 PMC6561910

[ref21] Li S.; Yu S.; Collins S. M.; Johnstone D. N.; Ashling C. W.; Sapnik A. F.; Chater P. A.; Keeble D. S.; McHugh L. N.; Midgley P. A.; Keen D. A.; Bennett T. D. A New Route to Porous Metal–Organic Framework Crystal–Glass Composites. Chem. Sci. 2020, 11, 9910–9918. 10.1039/d0sc04008h.

[ref22] Loiseau T.; Serre C.; Huguenard C.; Fink G.; Taulelle F.; Henry M.; Bataille T.; Férey G. A Rationale for the Large Breathing of the Porous Aluminum Terephthalate (MIL-53) Upon Hydration. Chem. - Eur. J. 2004, 10, 1373–1382. 10.1002/chem.200305413. 15034882

[ref23] Volkringer C.; Loiseau T.; Guillou N.; Fèrey G.; Haouas M.; Taulelle F.; Audebrand N.; Margiolaki I.; Popov D.; Burghammer M.; Riekel C. Structural Transitions and Flexibility during Dehydration - Rehydration Process in the MOF-Type Aluminum Pyromellitate A1_2_(OH)_2_[C_10_O_8_H_2_](MIL-118). Cryst. Growth Des. 2009, 9, 2927–2936. 10.1021/cg900276g.

[ref24] Banerjee D.; Kim S. J.; Parise J. B. Lithium Based Metal–Organic Framework with Exceptional Stability. Cryst. Growth Des. 2009, 9, 2500–2503. 10.1021/cg8014157.

[ref25] Moghadam P. Z.; Li A.; Liu X. W.; Bueno-Perez R.; Wang S. D.; Wiggin S. B.; Wood P. A.; Fairen-Jimenez D. Targeted Classification of Metal-Organic Frameworks in the Cambridge Structural Database (CSD). Chem. Sci. 2020, 11, 8373–8387. 10.1039/d0sc01297a. 33384860 PMC7690317

[ref26] Ashling C. W.; Johnstone D. N.; Widmer R. N.; Hou J.; Collins S. M.; Sapnik A. F.; Bumstead A. M.; Midgley P. A.; Chater P. A.; Keen D. A.; Bennett T. D. Synthesis and Properties of a Compositional Series of MIL-53(Al) Metal-Organic Framework Crystal-Glass Composites. J. Am. Chem. Soc. 2019, 141, 15641–15648. 10.1021/jacs.9b07557. 31491080 PMC7007233

[ref27] Krishnan R. S.; Srinivasan R.; Devanarayanan S. Thermal Expansion of Crystals, First; Pergamon Press Ltd., 1979.

[ref28] Nanthamathee C.; Ling S.; Slater B.; Attfield M. P. Contradistinct Thermoresponsive Behavior of Isostructural MIL-53 Type Metal-Organic Frameworks by Modifying the Framework Inorganic Anion. Chem. Mater. 2015, 27, 85–95. 10.1021/cm503311x.

[ref29] Cabañero J. M.; Pimenta V.; Cannon K. C.; Morris R. E.; Armstrong A. R. Sodium Naphthalene-2,6-Dicarboxylate: An Anode for Sodium Batteries. ChemSusChem 2019, 12, 4522–4528. 10.1002/cssc.201901626. 31403248

[ref30] Coelho A. A. TOPAS and TOPAS-Academic: An Optimization Program Integrating Computer Algebra and Crystallographic Objects Written in C++. J. Appl. Crystallogr. 2018, 51, 210–218. 10.1107/S1600576718000183.

[ref31] Coelho A. A. TOPAS Academic Version 6 (Computer Software); Coelho Software: Brisbane, 2016.

[ref32] Yim W. M.; Paff R. J. Thermal Expansion of AlN, Sapphire, and Silicon. J. Appl. Phys. 1974, 45, 1456–1457. 10.1063/1.1663432.

[ref33] Cliffe M. J.; Hill J. A.; Murray C. A.; Coudert F. X.; Goodwin A. L. Defect-Dependent Colossal Negative Thermal Expansion in UiO-66(Hf) Metal-Organic Framework. Phys. Chem. Chem. Phys. 2015, 17, 11586–11592. 10.1039/c5cp01307k. 25866163

[ref34] Lindig O.; Pannhorst W. Thermal Expansion and Length Stability of Zerodur in Dependence on Temperature and Time. Appl. Opt. 1985, 24, 3330–3334. 10.1364/AO.24.003330. 18224051

