# Supplementary material for: Thermal Expansion of Metal–Organic Framework Crystal–Glass Composites
Source: Inorg Chem. 2022 Nov 8;61(46):18458–65. doi: 10.1021/acs.inorgchem.2c02663 (PMC9682478; doi:10.1021/acs.inorgchem.2c02663)
Supplement: Supplementary file 1 — ic2c02663_si_001.pdf [file ic2c02663_si_001.pdf]

Supporting Information

Thermal Expansion of Metal–Organic Framework Crystal–Glass Composites

Christopher W. Ashling[1], Giulio I. Lampronti[2], Thomas J. F. Southern[1], Rachel C. Evans[1], and Thomas D. Bennett[1*]

[1]. Department of Materials Science and Metallurgy, University of Cambridge, Cambridge, CB3 0FS, U.K.
[2]. Department of Earth Sciences, University of Cambridge, Cambridge, CB2 3EQ, U.K.

Email: tdb35@cam.ac.uk

Contents:

1. Powder X-ray Diffraction
2. Variable Temperature Powder X-ray Diffraction
3. Thermal Expansion
4. VT-PXRD Refinement Values

1. Powder X-ray Diffraction Data

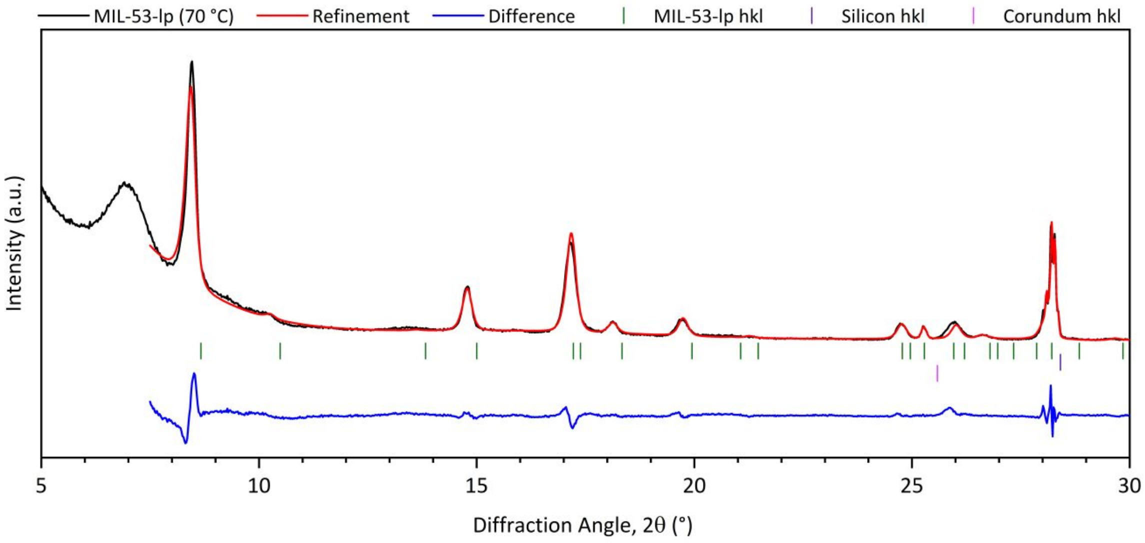

Figure S1. Initial refinement of MIL-53-lp at 70 °C with peak marks for all phases.

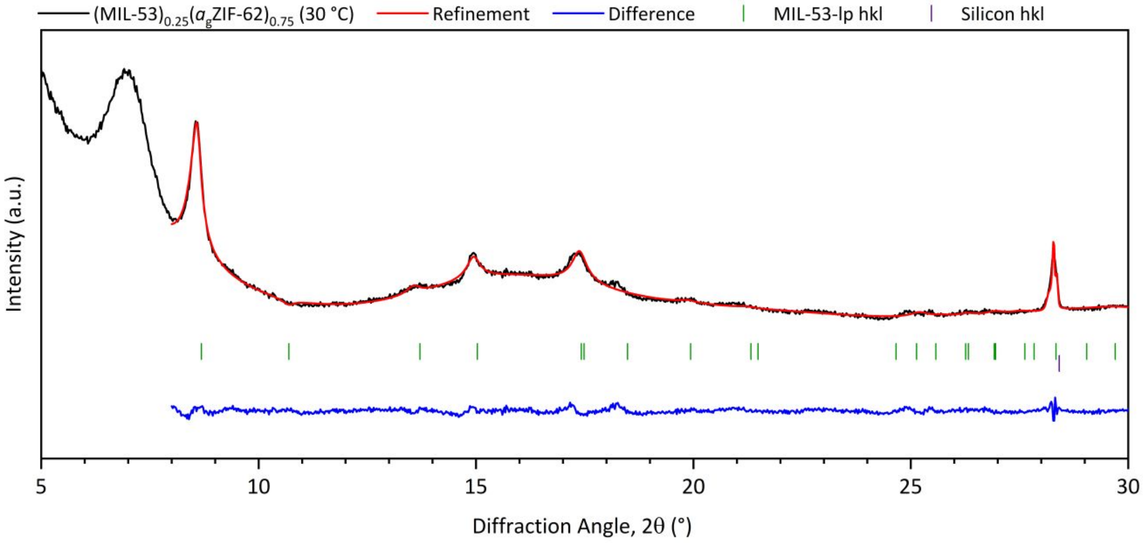

Figure S2. Initial refinement of $(MIL\text{-}53)_{0.25}(a_gZIF\text{-}62)_{0.75}$ with peak marks for all phases.

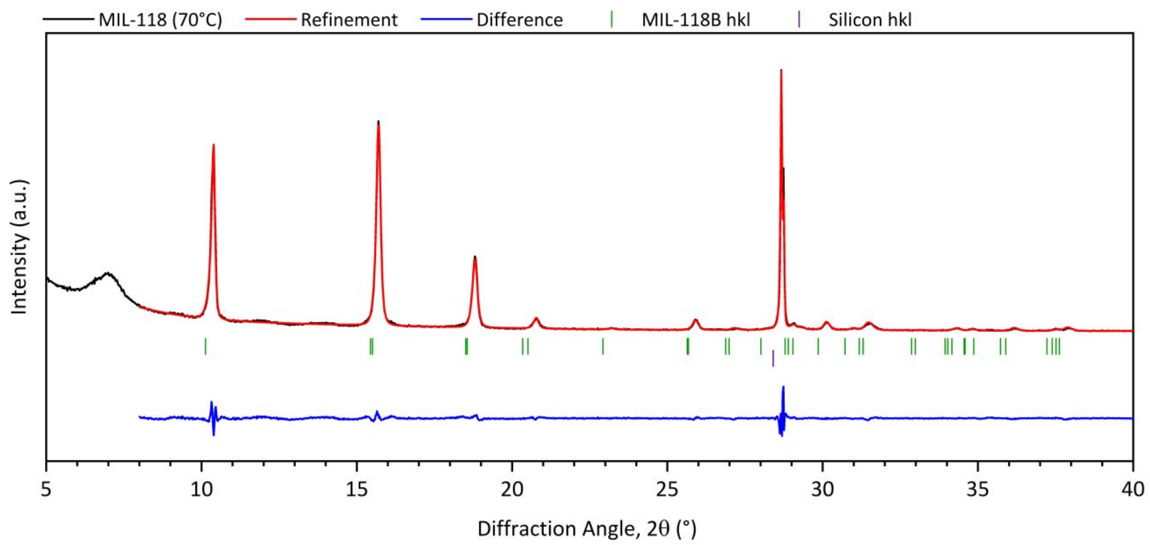

Figure S3. Initial refinement of MIL-118 with peak marks for all phases.

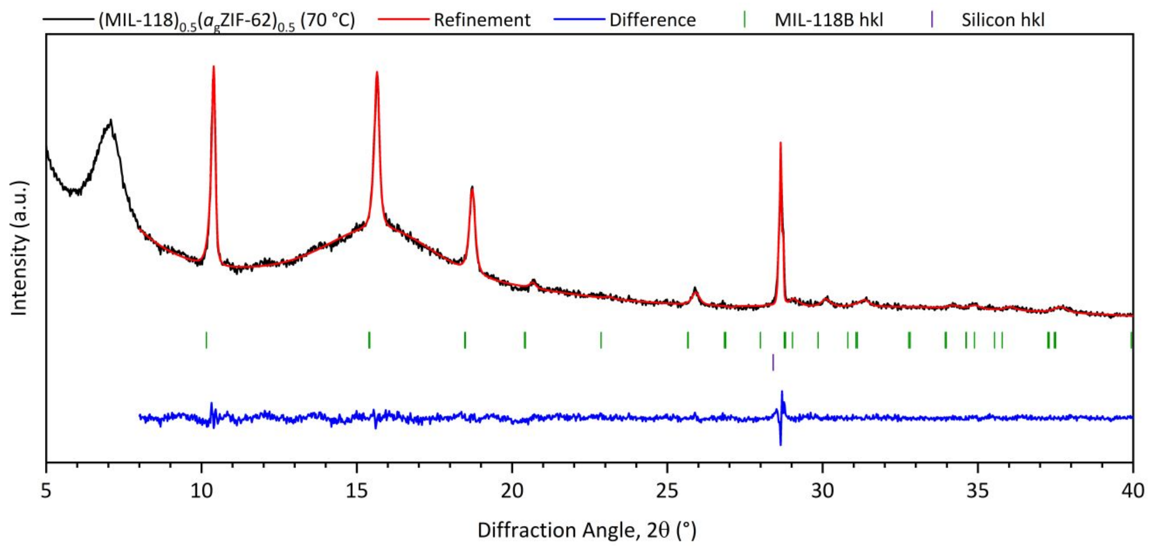

Figure S4. Initial refinement of $(MIL\text{-}118)_{0.5}(a_gZIF\text{-}62)_{0.5}$ with peak marks for all phases.

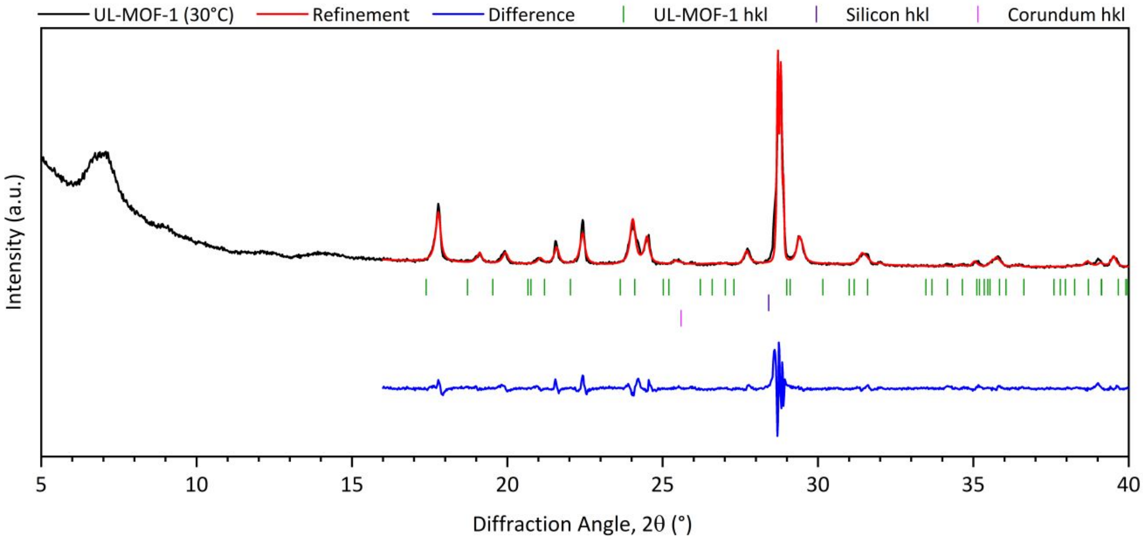

Figure S5. Initial refinement of UL-MOF-1 with peak marks for all phases.

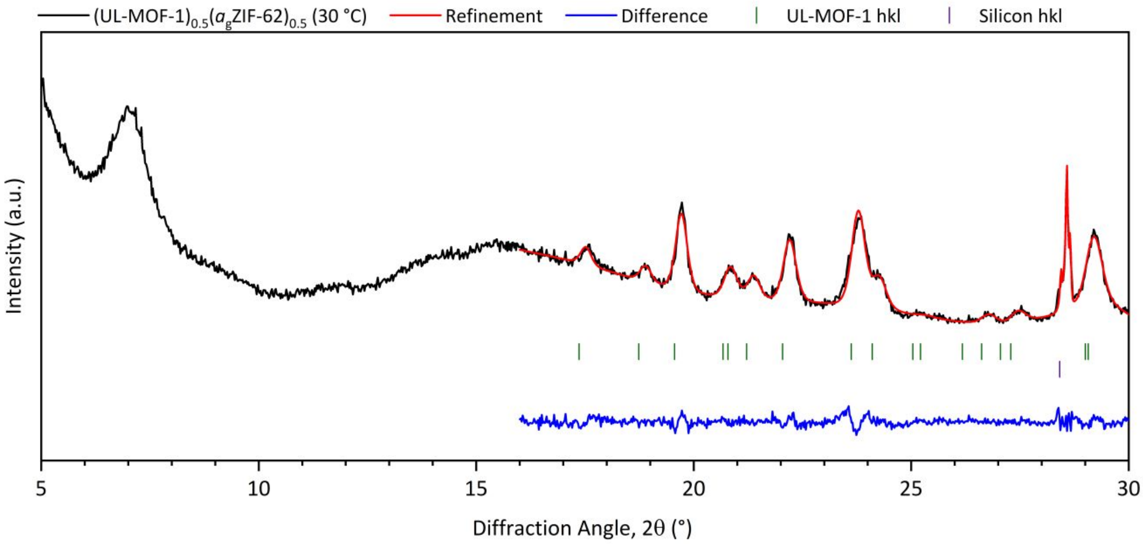

Figure S6. Initial refinement of $(UL\text{-}MOF\text{-}1)_{0.5}(a_gZIF\text{-}62)_{0.5}$ with peak marks for all phases.

2. Variable Temperature Powder X-ray Diffraction

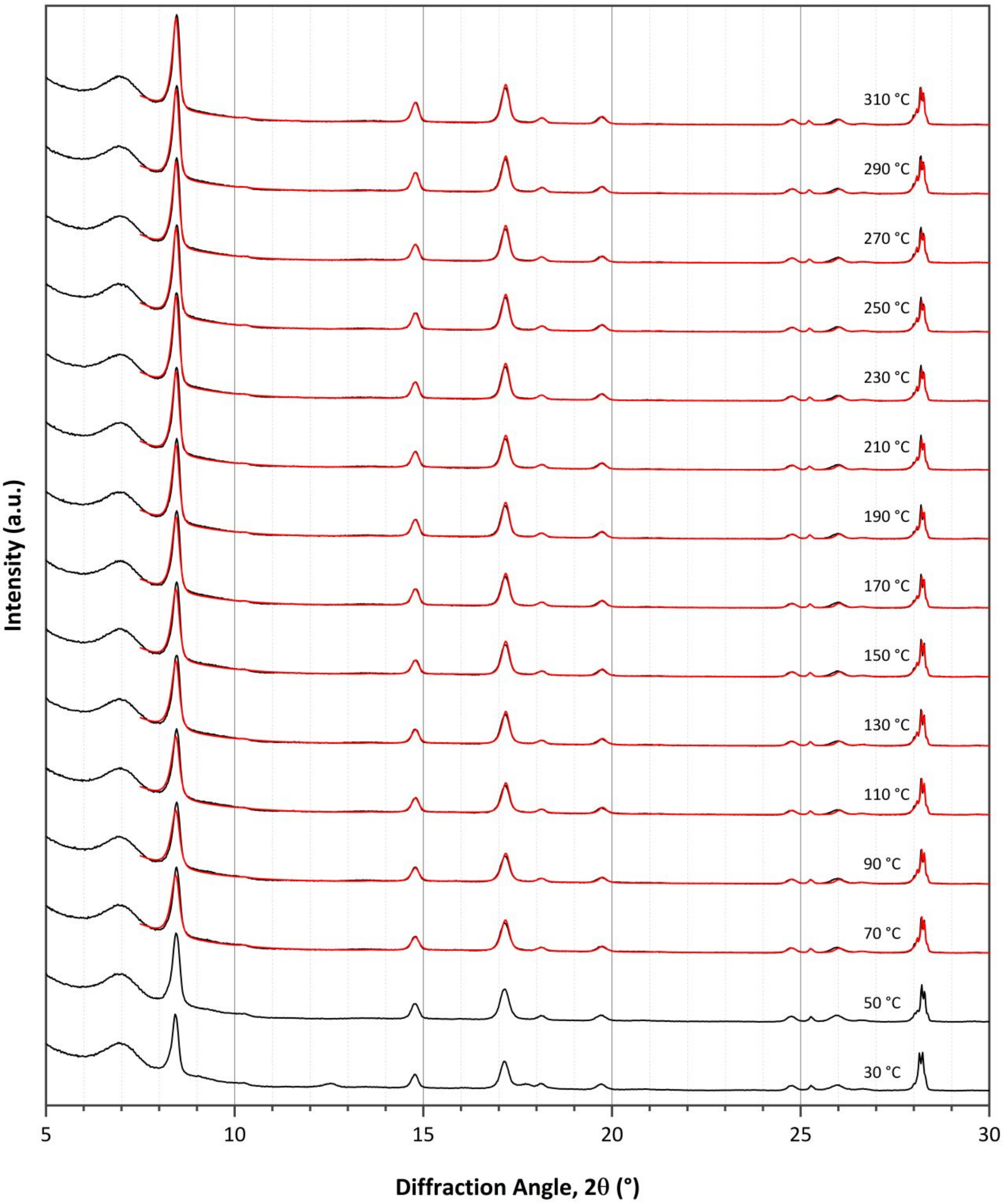

Figure S7. Variable temperature powder X-ray diffraction patterns of MIL-53-lp. Black – experimental data. Red - Refinement.

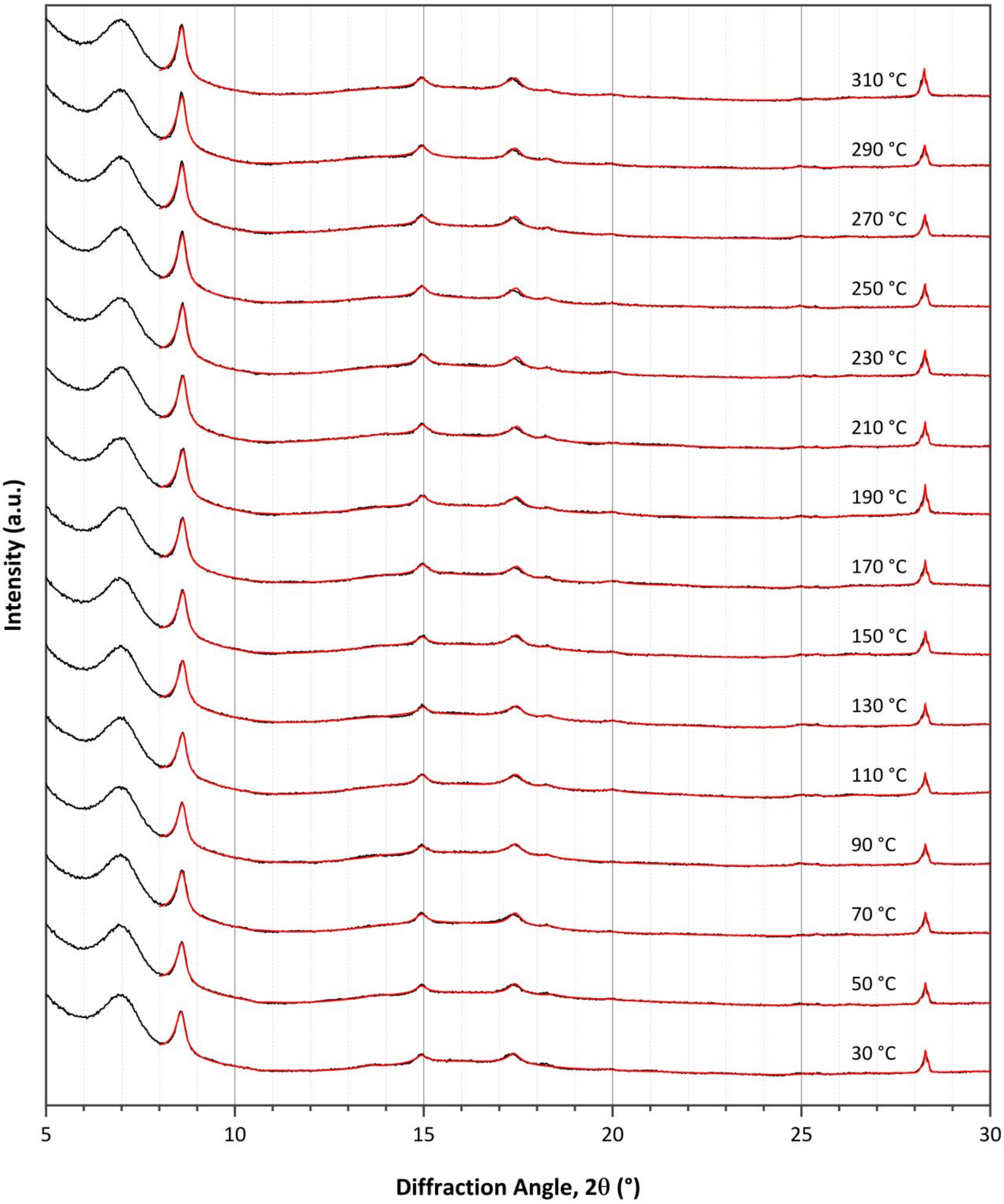

Figure S8. Variable temperature powder X-ray diffraction patterns of $(MIL\text{-}53)_{0.25}(a_g ZIF\text{-}62)_{0.75}$. Black – experimental data. Red - Refinement.

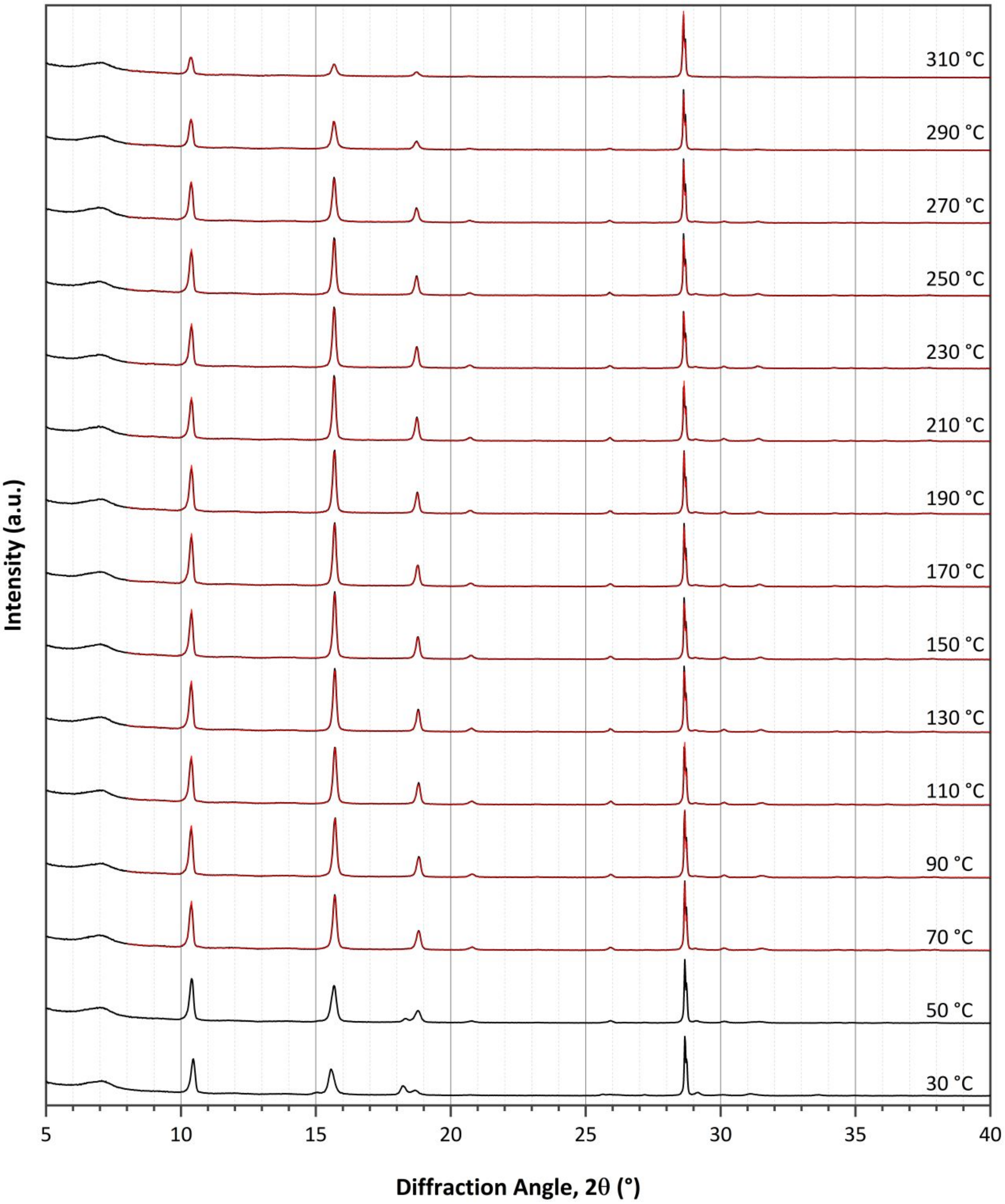

Figure S9. Variable temperature powder X-ray diffraction patterns of MIL-118. Black – experimental data. Red - Refinement.

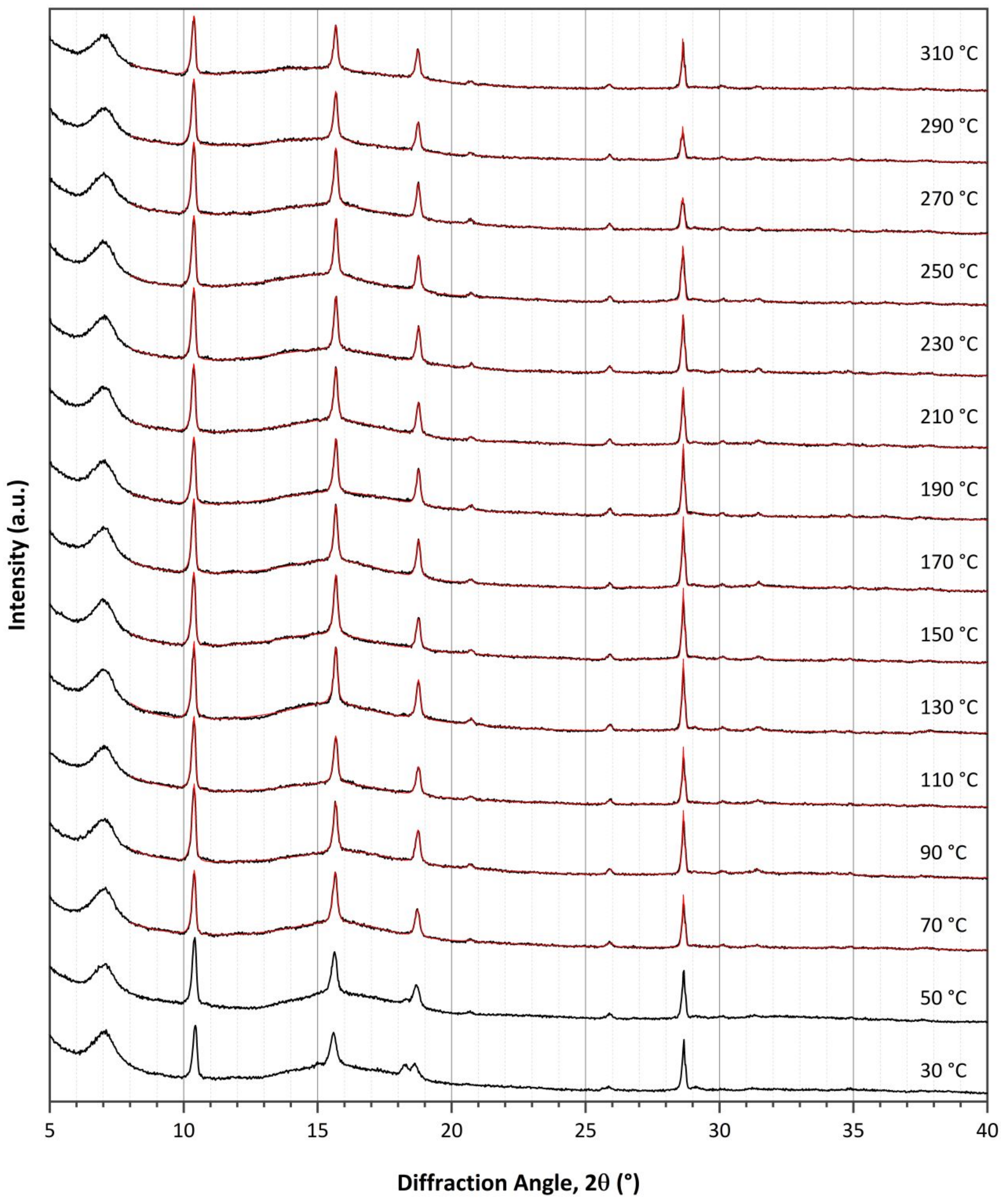

Figure S10. Variable temperature powder X-ray diffraction patterns of $(MIL\text{-}118)_{0.5}(a_{g}ZIF\text{-}62)_{0.5}$. Black – experimental data. Red - Refinement.

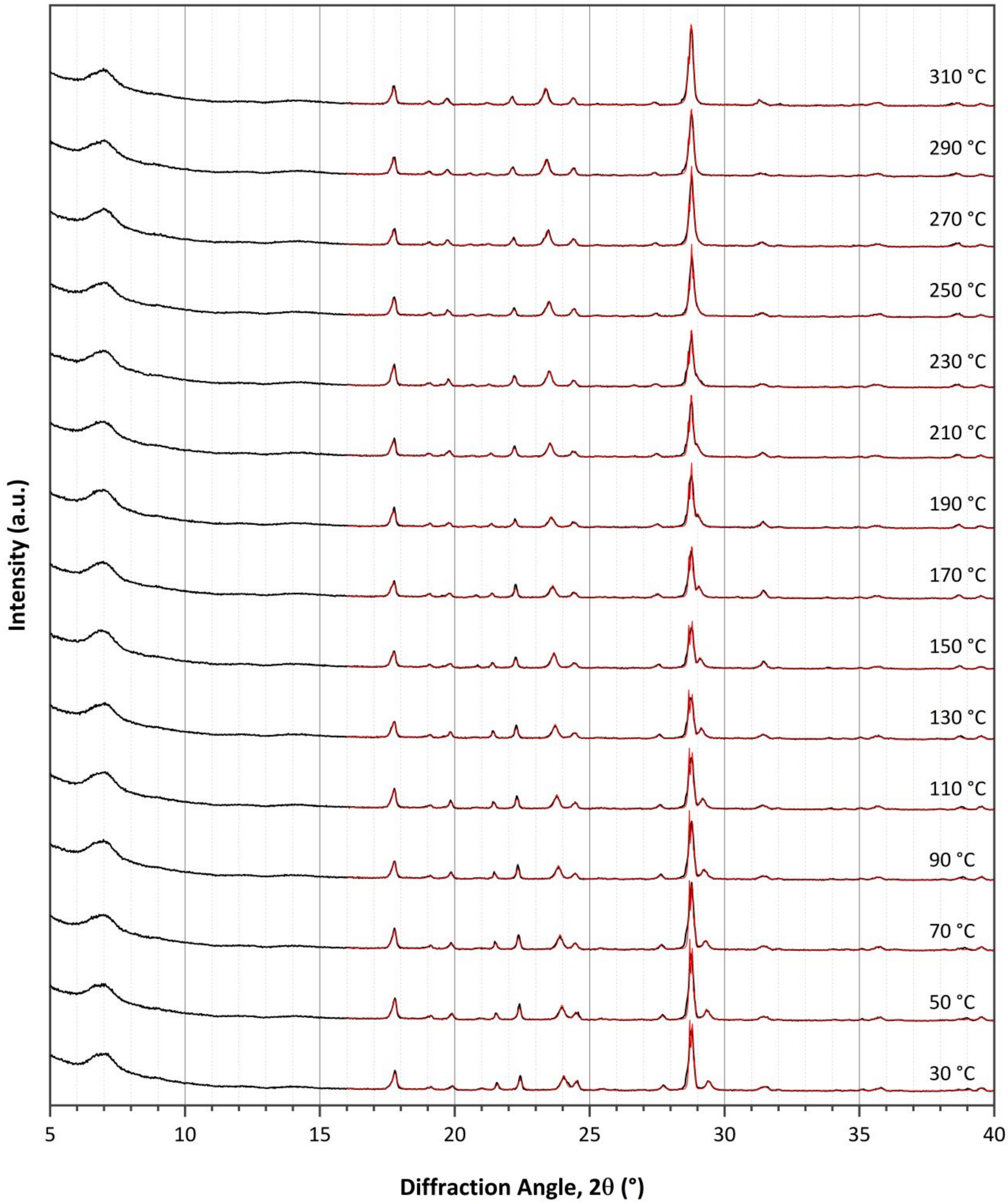

Figure S11. Variable temperature powder X-ray diffraction patterns of UL-MOF-1. Black – experimental data. Red - Refinement.

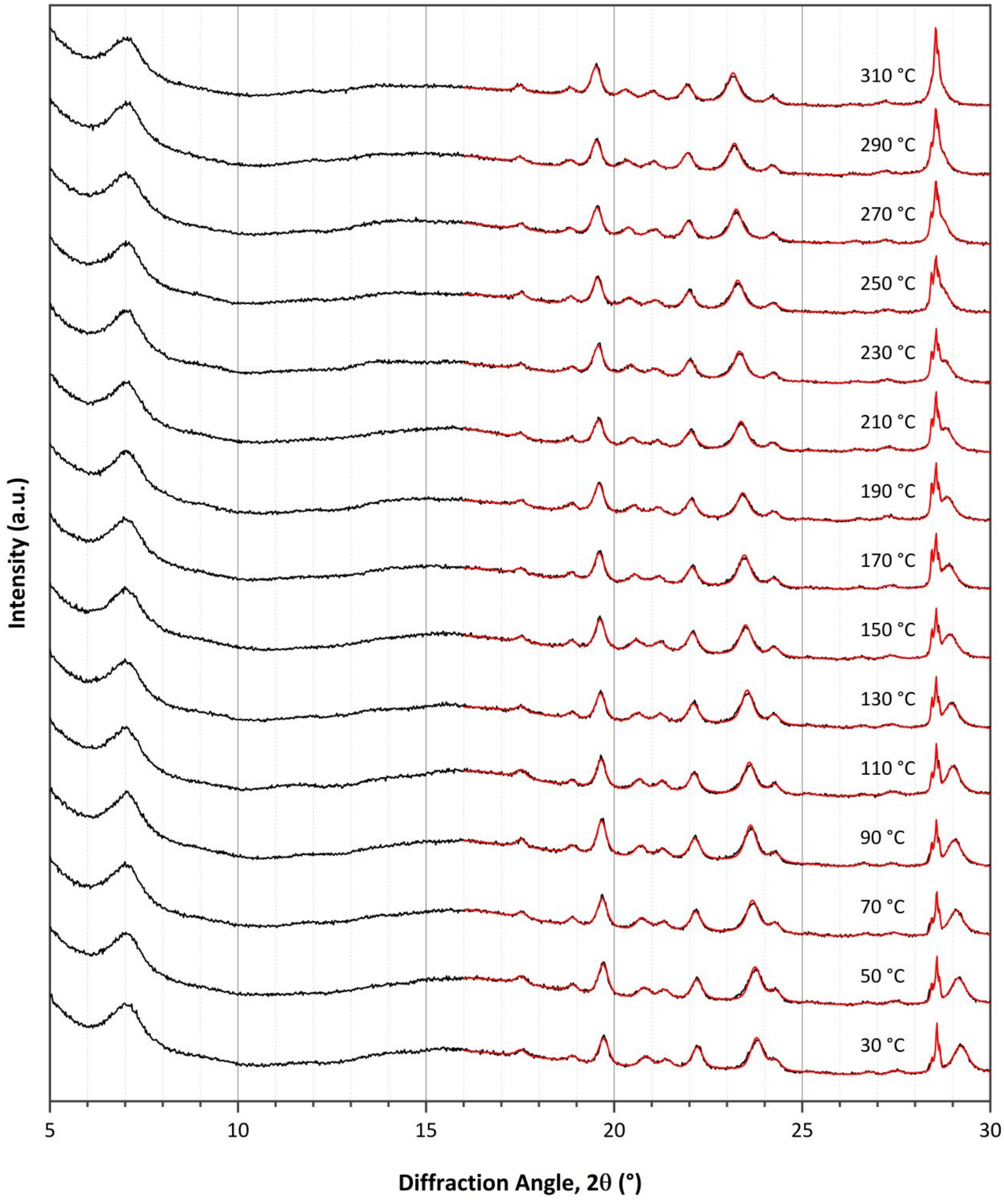

Figure S12. Variable temperature powder X-ray diffraction patterns of (UL-MOF-1)$_{0.5}$(a_gZIF-62)$_{0.5}$. Black – experimental data. Red - Refinement.

3. Thermal Expansion

The coefficient of thermal expansion is defined by **Equation S1**:

$$\alpha_a = \frac{1}{a_0}\left(\frac{da}{dT}\right)_P$$

Assuming α_a is constant with temperature allows integration of S1 to give **Equation S2**:

$$\Delta a = a_0\left(e^{\alpha_a \Delta T} - 1\right)$$

Which has a Taylor expansion of **Equation S3**:

$$\Delta a = a_0\left(\left(1 + \alpha_a \Delta T + \frac{\alpha_a^2 \Delta T^2}{2} + \ldots\right) - 1\right)$$

The value of $\frac{a_0 \alpha_a^2 \Delta T^2}{2}$ is comparable to the error in the lattice parameter measurement for all samples such that the Taylor expansion was truncated after the second term giving **Equation 2** in the manuscript:

$$\alpha_a = \frac{1}{a_0}\left(\frac{\Delta a}{\Delta T}\right)_P$$

4. VTPXRD Refinement Values

Table S1. Refinement values of the VT-PXRD of MIL-53-lp

T (°C)	rwp	χ^2	*a* (Å)	*a* st. dev.	*b* (Å)	*b* st. dev.	*c* (Å)	*c* st. dev.	Volume (Å³)	Volume st. dev.
**70**	9.71	4.04	6.6494	0.0019	16.8596	0.0143	12.7927	0.0066	1434.15	1.48
**90**	10.07	4.11	6.6498	0.0019	16.8513	0.0150	12.7879	0.0069	1432.97	1.54
**110**	9.61	3.96	6.6485	0.0018	16.8548	0.0139	12.7840	0.0063	1432.55	1.43
**130**	9.46	3.90	6.6489	0.0017	16.8496	0.0130	12.7872	0.0060	1432.57	1.35
**150**	9.40	3.92	6.6490	0.0017	16.8386	0.0127	12.7952	0.0059	1432.54	1.32
**170**	9.38	3.86	6.6499	0.0016	16.8381	0.0126	12.7996	0.0059	1433.20	1.30
**190**	9.24	3.79	6.6510	0.0015	16.8337	0.0118	12.8065	0.0056	1433.84	1.23
**210**	9.28	3.78	6.6516	0.0016	16.8328	0.0122	12.8007	0.0058	1433.23	1.27
**230**	9.28	3.82	6.6524	0.0015	16.8406	0.0118	12.8015	0.0055	1434.16	1.23
**250**	8.97	3.69	6.6514	0.0015	16.8323	0.0112	12.8078	0.0053	1433.93	1.17
**270**	9.24	3.78	6.6519	0.0015	16.8450	0.0116	12.8014	0.0054	1434.41	1.20
**290**	9.14	3.81	6.6511	0.0014	16.8282	0.0110	12.8054	0.0052	1433.26	1.15
**310**	8.81	3.73	6.6512	0.0014	16.8295	0.0106	12.8055	0.0050	1433.38	1.10

Table S2. Refinement values of the VT-PXRD of $(MIL\text{-}53)_{0.25}(a_gZIF\text{-}62)_{0.75}$

T (°C)	rwp	χ^2	*a* (Å)	*a* st. dev.	*b* (Å)	*b* st. dev.	*c* (Å)	*c* st. dev.	Volume (Å³)	Volume st. dev.
**30**	2.95	1.63	6.5848	0.0051	16.2963	0.0246	12.9001	0.0123	1384.29	2.70
**50**	2.91	1.60	6.6003	0.0046	16.4249	0.0264	12.7947	0.0128	1387.06	2.79
**70**	3.12	1.71	6.6236	0.0041	16.7346	0.0320	12.6471	0.0144	1401.85	3.24
**90**	2.98	1.63	6.6269	0.0046	16.8022	0.0270	12.5993	0.0113	1402.89	2.76
**110**	2.85	1.55	6.6281	0.0035	16.8297	0.0239	12.5649	0.0098	1401.60	2.39
**130**	3.02	1.65	6.6176	0.0043	16.8210	0.0262	12.5668	0.0110	1398.88	2.66
**150**	2.94	1.61	6.6212	0.0042	16.7489	0.0261	12.6057	0.0113	1397.93	2.67
**170**	3.16	1.74	6.6201	0.0041	16.8457	0.0342	12.5637	0.0137	1401.10	3.34
**190**	3.43	1.88	6.6183	0.0044	16.7338	0.0388	12.6074	0.0165	1396.27	3.83
**210**	2.98	1.66	6.6251	0.0035	16.8340	0.0254	12.5623	0.0104	1401.04	2.52
**230**	3.17	1.76	6.6193	0.0040	16.8359	0.0379	12.5660	0.0154	1400.38	3.69
**250**	3.05	1.68	6.6226	0.0034	16.8532	0.0323	12.5795	0.0134	1404.01	3.16
**270**	3.08	1.71	6.6192	0.0040	16.7919	0.0364	12.6243	0.0155	1403.18	3.60
**290**	3.21	1.78	6.6198	0.0037	16.8022	0.0332	12.6268	0.0143	1404.43	3.29
**310**	3.04	1.67	6.6160	0.0034	16.7874	0.0239	12.6114	0.0100	1400.68	2.39

Table S3. Refinement values of the VT-PXRD of MIL-118

T (°C)	rwp	χ^2	*a* (Å)	*a* st. dev.	*b* (Å)	*b* st. dev.	*c* (Å)	*c* st. dev.	Volume (Å³)	Volume st. dev.
**70**	8.42	2.03	11.4201	0.0015	6.6282	0.0005	8.7238	0.0007	660.35	0.11
**90**	8.19	1.99	11.4128	0.0013	6.6248	0.0004	8.7258	0.0007	659.73	0.10
**110**	8.37	2.02	11.4220	0.0013	6.6221	0.0004	8.7210	0.0007	659.63	0.10
**130**	8.24	2.00	11.4321	0.0012	6.6222	0.0004	8.7193	0.0006	660.10	0.09
**150**	8.39	2.02	11.4492	0.0011	6.6211	0.0004	8.7149	0.0006	660.65	0.09
**170**	8.38	2.02	11.4584	0.0011	6.6207	0.0004	8.7137	0.0006	661.05	0.09
**190**	8.30	1.99	11.4695	0.0011	6.6212	0.0004	8.7117	0.0006	661.59	0.09
**210**	8.64	2.08	11.4835	0.0011	6.6207	0.0004	8.7096	0.0007	662.18	0.09
**230**	8.84	2.10	11.4914	0.0011	6.6192	0.0004	8.7074	0.0007	662.31	0.10
**250**	8.63	2.06	11.4925	0.0013	6.6175	0.0004	8.7073	0.0007	662.20	0.10
**270**	8.08	1.90	11.4860	0.0015	6.6175	0.0005	8.7108	0.0008	662.10	0.12
**290**	7.59	1.74	11.4868	0.0024	6.6175	0.0006	8.7125	0.0009	662.27	0.17
**310**	6.57	1.48	11.4776	0.0032	6.6135	0.0009	8.7087	0.0010	661.05	0.22

Table S4. Refinement values of the VT-PXRD of $(MIL\text{-}118)_{0.5}(a_gZIF\text{-}62)_{0.5}$

T (°C)	rwp	χ²	*a* (Å)	*a* st. dev.	*b* (Å)	*b* st. dev.	*c* (Å)	*c* st. dev.	Volume (Å³)	Volume st. dev.
**70**	4.03	1.33	11.5017	0.0025	6.6281	0.0010	8.6987	0.0010	663.15	0.19
**90**	4.17	1.42	11.4791	0.0018	6.6248	0.0010	8.7036	0.0009	661.88	0.16
**110**	4.17	1.36	11.4602	0.0026	6.6252	0.0012	8.7087	0.0009	661.21	0.20
**130**	4.75	1.65	11.4583	0.0023	6.6251	0.0011	8.7102	0.0011	661.21	0.19
**150**	4.16	1.43	11.4583	0.0024	6.6234	0.0009	8.7115	0.0009	661.14	0.18
**170**	4.09	1.42	11.4537	0.0021	6.6259	0.0010	8.7114	0.0010	661.12	0.17
**190**	4.23	1.45	11.4537	0.0025	6.6242	0.0009	8.7104	0.0010	660.87	0.19
**210**	3.80	1.28	11.4525	0.0022	6.6246	0.0009	8.7140	0.0008	661.12	0.17
**230**	4.18	1.43	11.4556	0.0021	6.6219	0.0010	8.7123	0.0010	660.89	0.17
**250**	4.21	1.44	11.4564	0.0023	6.6207	0.0009	8.7106	0.0010	660.69	0.18
**270**	4.02	1.38	11.4657	0.0019	6.6209	0.0008	8.7119	0.0010	661.35	0.15
**290**	4.08	1.30	11.4714	0.0020	6.6228	0.0008	8.7115	0.0009	661.84	0.16
**310**	4.57	1.44	11.4778	0.0024	6.6222	0.0010	8.7114	0.0011	662.14	0.19

Table S5. Refinement values of the VT-PXRD of UL-MOF-1

T (°C)	rwp	χ²	*a* (Å)	*a* st. dev.	*b* (Å)	*b* st. dev.	*c* (Å)	*c* st. dev.	*β* (°)	*β* st. dev.	Volume (Å³)	Volume st. dev.
**30**	15.63	2.87	10.3062	0.0025	5.3492	0.0016	8.6859	0.0021	98.5935	0.0006	473.48	0.22
**50**	15.12	2.81	10.3077	0.0025	5.3545	0.0015	8.7117	0.0020	98.5306	0.0006	475.50	0.21
**70**	16.06	2.98	10.3120	0.0026	5.3581	0.0016	8.7322	0.0019	98.3901	0.0006	477.31	0.21
**90**	14.26	2.60	10.3115	0.0027	5.3593	0.0015	8.7509	0.0018	98.2870	0.0006	478.55	0.21
**110**	13.69	2.50	10.3123	0.0024	5.3610	0.0013	8.7674	0.0017	98.1874	0.0006	479.76	0.19
**130**	13.71	2.48	10.3098	0.0026	5.3604	0.0014	8.7821	0.0018	98.0796	0.0006	480.52	0.20
**150**	13.01	2.32	10.3110	0.0024	5.3630	0.0015	8.7926	0.0018	97.9552	0.0006	481.53	0.20
**170**	13.53	2.43	10.3124	0.0026	5.3614	0.0015	8.8049	0.0020	97.8849	0.0006	482.21	0.21
**190**	13.43	2.41	10.3089	0.0048	5.3647	0.0022	8.8227	0.0027	97.8675	0.0010	483.34	0.33
**210**	12.73	2.31	10.3123	0.0025	5.3635	0.0014	8.8384	0.0023	97.8394	0.0006	484.28	0.21
**230**	12.46	2.27	10.3104	0.0022	5.3634	0.0013	8.8582	0.0023	97.8873	0.0006	485.21	0.20
**250**	11.59	2.10	10.3117	0.0021	5.3726	0.0013	8.8686	0.0021	97.8240	0.0006	486.75	0.19
**270**	12.18	2.23	10.3119	0.0034	5.3773	0.0019	8.8836	0.0031	97.7801	0.0008	488.06	0.29
**290**	11.54	2.11	10.3111	0.0040	5.3801	0.0022	8.9038	0.0035	97.7649	0.0010	489.41	0.34
**310**	11.56	2.19	10.3106	0.0052	5.3821	0.0029	8.9135	0.0046	97.7127	0.0013	490.16	0.44

Table S6. Refinement values of the VT-PXRD of $(UL\text{-}MOF\text{-}1)_{0.5}(a_gZIF\text{-}62)_{0.5}$

T (°C)	rwp	χ²	*a* (Å)	*a* st. dev.	*b* (Å)	*b* st. dev.	*c* (Å)	*c* st. dev.	*β* (°)	*β* st. dev.	Volume (Å³)	Volume st. dev.
**30**	4.16	1.50	10.3176	0.0058	5.3405	0.0011	8.6812	0.0026	98.4889	0.0009	473.10	0.32
**50**	4.29	1.57	10.3200	0.0055	5.3414	0.0011	8.7025	0.0026	98.5213	0.0009	474.41	0.31
**70**	3.94	1.41	10.3059	0.0057	5.3437	0.0011	8.7229	0.0023	98.3941	0.0009	475.24	0.31
**90**	4.09	1.51	10.3095	0.0055	5.3447	0.0011	8.7352	0.0025	98.3061	0.0009	476.27	0.31
**110**	3.98	1.45	10.3004	0.0048	5.3482	0.0011	8.7433	0.0021	98.1934	0.0008	476.74	0.27
**130**	4.10	1.45	10.3097	0.0059	5.3482	0.0011	8.7620	0.0026	98.1571	0.0010	478.24	0.33
**150**	3.56	1.28	10.3005	0.0052	5.3504	0.0010	8.7733	0.0021	98.0308	0.0008	478.77	0.28
**170**	3.92	1.39	10.3084	0.0062	5.3536	0.0011	8.7911	0.0027	98.0782	0.0010	480.34	0.34
**190**	3.68	1.27	10.3049	0.0052	5.3548	0.0010	8.7990	0.0023	97.9314	0.0008	480.89	0.29
**210**	3.85	1.32	10.3136	0.0063	5.3564	0.0011	8.8134	0.0028	97.8776	0.0010	482.29	0.35
**230**	3.79	1.30	10.3142	0.0057	5.3564	0.0011	8.8343	0.0026	97.8618	0.0009	483.48	0.32
**250**	4.26	1.47	10.2976	0.0059	5.3599	0.0012	8.8478	0.0031	97.7407	0.0010	483.90	0.35
**270**	4.01	1.40	10.3000	0.0061	5.3607	0.0011	8.8537	0.0027	97.6302	0.0010	484.53	0.34
**290**	4.08	1.39	10.3062	0.0058	5.3635	0.0012	8.8694	0.0032	97.5698	0.0010	486.01	0.34
**310**	3.99	1.38	10.3049	0.0056	5.3643	0.0011	8.8846	0.0032	97.5437	0.0010	486.88	0.34

Table S7. Volumetric changes of MIL-53 and MIL-118 from their high- to low-temperature phases.

	**MIL-53**			**MIL-118**		
Axis	MIL-53-lp (Å)	MIL-53-np (Å)	Change (%)	MIL-118B (Å)	MIL-118C (Å)	Change (%)
x	16.675	19.513	17.02	8.722	8.614	- 1.23
y	12.813	7.612	- 40.59	5.666	6.067	7.08
z	3.304	3.288	0.48	3.312	3.208	-3.14

In the case of MIL-53, the *x* and *y* axes are equal to the distance between Al atoms corresponding to the width and height of the diamond-shaped pores. The *z* axis is equal to the Al–Al distance across the Al–O–Al bonds along the Al–O columns.

For MIL-118, the *x* and *y* axes are the distances between Al–O columns, and the *z* axis is the distance between Al–Al atoms within the Al–O columns.
